# Correction: Unravelling the mystery of “Madagascar copal”: Age, origin and preservation of a Recent resin

**DOI:** 10.1371/journal.pone.0235695

**Published:** 2020-07-02

**Authors:** The PLOS ONE Staff

Fig 3 appears in black and white rather than in color. Please see the correct Fig 3 here. The publisher apologizes for the error.

**Fig 3:**
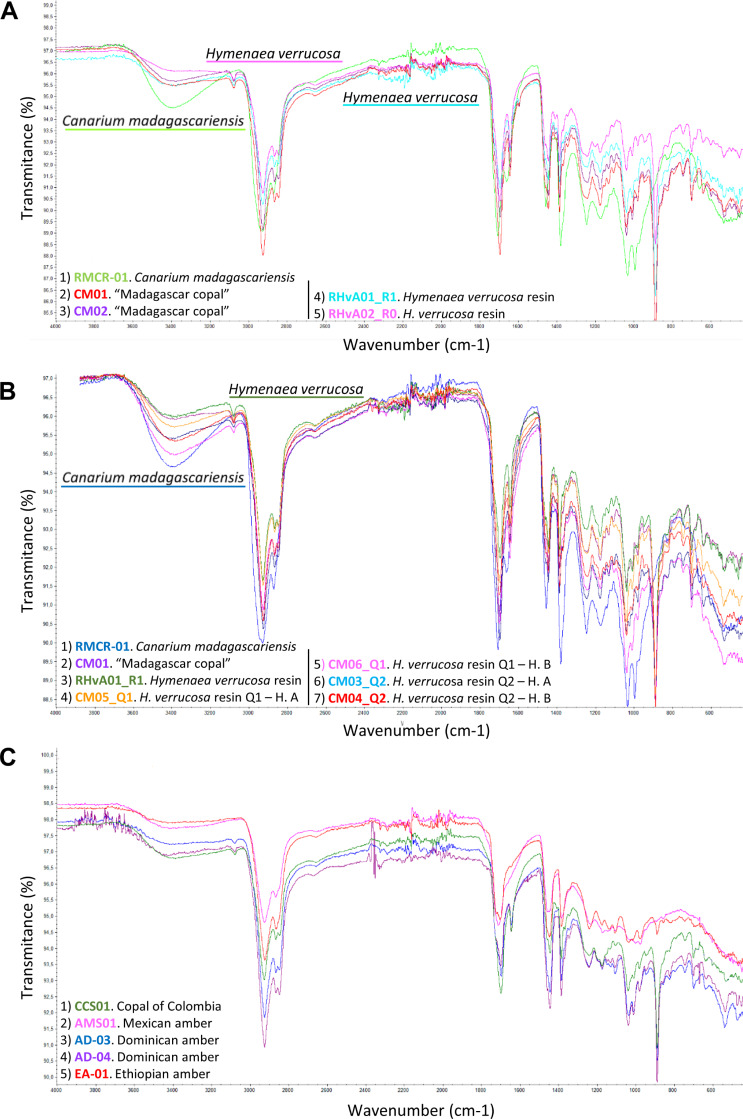
FTIR analyses comparing the differences between resin, copal and amber produced by *Hymenaea* spp. and *Canarium madagascariensis*. (A) FTIR analyses of the samples in order to identify the tree-resin producer of “Madagascar copal”, include (see Table 1): *Canarium madagascariensis* and two samples of “Madagascar copal” (CM01 and CM02), two samples of resin from *H*. *verrucosa* branches (RHvA01-R1 and RHvA02-R0). (B) Malagasy *H*. *verrucosa* analyses: FTIR analyses that show the comparison between the results of A and other resin, copal and amber produced by *Hymenaea* ssp. The analyses include samples of *Canarium madagascariensis* (RMCR-01), “Madagascar copal” (CM01), resin from *H*. *verrucosa* branch, (RHvA01-R1), resin from *H*. *verrucosa* of the Andranotsara pit Q1, found in A horizon A (CM05-Q1) and in the sub-horizon B_1_ (CM06-Q1), and resin pieces of *H*. *verrucosa* of the Antampolo pit Q2, found in A horizon (CM03-Q2) and in sub-horizon B_1_ (CM4-Q2). (C) Neotropical *Hymenaea* spp. resin and amber analyses: resin from “copal of Colombia”, (CCS01), Miocene Mexican amber (AMS01), Miocene Dominican amber (AD-03) and (AD-04), and Miocene Ethiopian amber (EA-01). Diterpenic resin/copal has some characteristic vibrational group frequencies: characteristic is a low intensity of absorption band at 3080 cm^-1^ that is absent from triterpenoid resin/copal and that corresponds to v (= C-H), intensity absorption band at 2937–2929 cm^-1^ corresponds to v_as_(C-H), CH_3_, CH_2_ (methylene group), intensity band at 2874–2844 cm^-1^ corresponds to v_s_ (C-H), CH_3_, CH_2_ (methyl group), intensity bands at 1718 cm^-1^, 1694 cm^-1^, and 1644 cm^-1^ correspond to v (C = O), intensity band at 1446 cm^-1^ corresponds to δ_as_ (CH_3_), intensity band at 1386 cm^-1^ corresponds to δ_s_ (CH_3_), and intensity band at 888 cm^-1^ corresponds out of plane δ (CH_2_) of the exomethylene functionality C_8_-C_20_. “Madagascar copal” and “East African Copal” can be differentiated from “Western African Copal” by the linear slope of the spectra in the case of the resin/copal of West Africa and the intensity of 3411–3422 cm^-1^ that corresponds to v (OH) of the East African copal. It is possible to differentiate between amber and copal by observing the exocyclic methylene bands at 3048, 1642 and 887 cm^-1^. In the case of copal, the first two bands are not intense, but they are clearly observed, and the band of 887 cm^-1^ is very intense. In the case of ambers, the bands are absent or of very weak intensity.
