# Supplementary figures and images for: Unravelling the mystery of “Madagascar copal”: Age, origin and preservation of a Recent resin
Source: PLoS One. 2020 May 18;15(5):e0232623. doi: 10.1371/journal.pone.0232623 (PMC7233546; doi:10.1371/journal.pone.0232623)

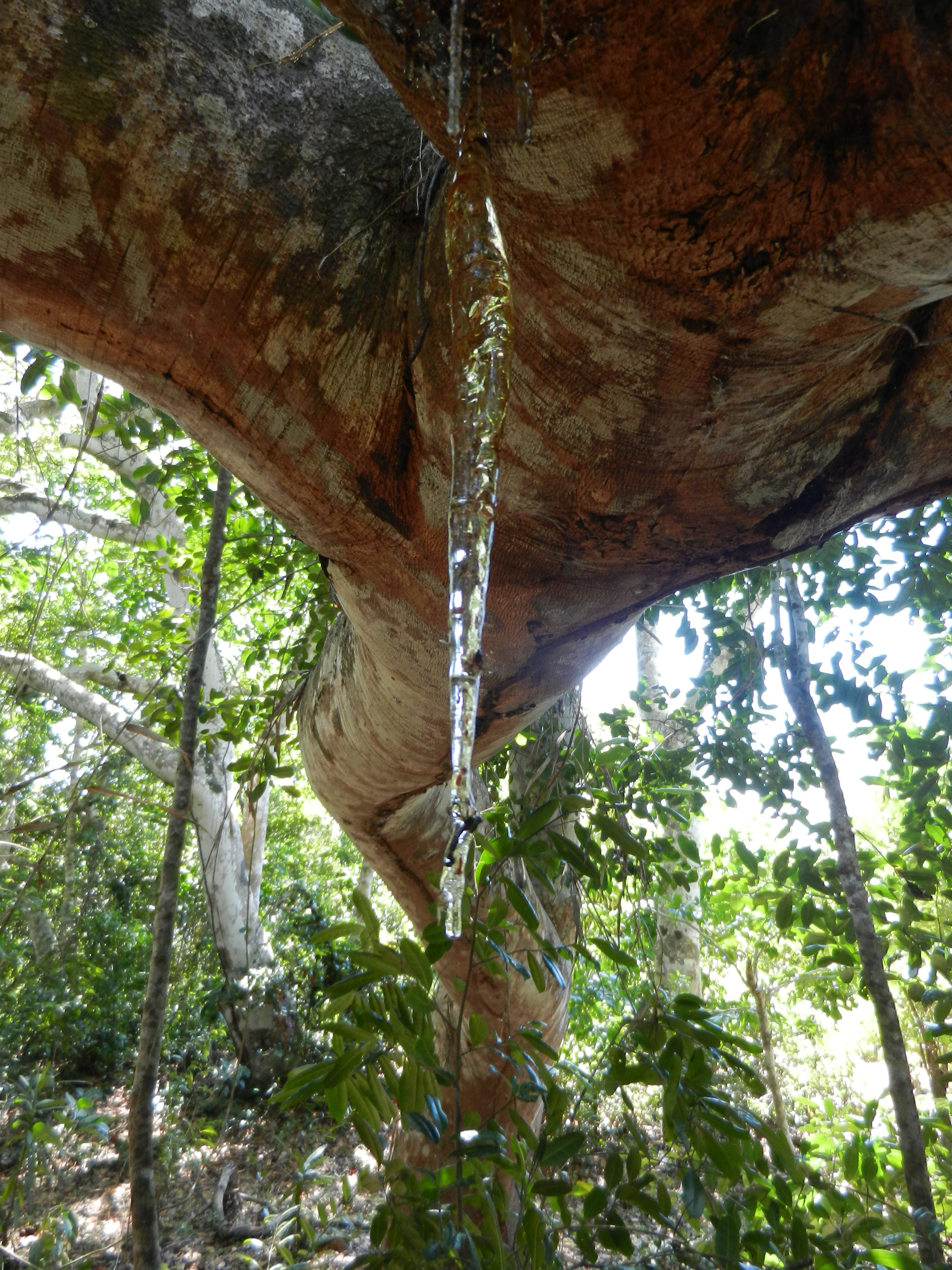

Supplement: S1 Fig — (TIF) [file pone.0232623.s001.tif]

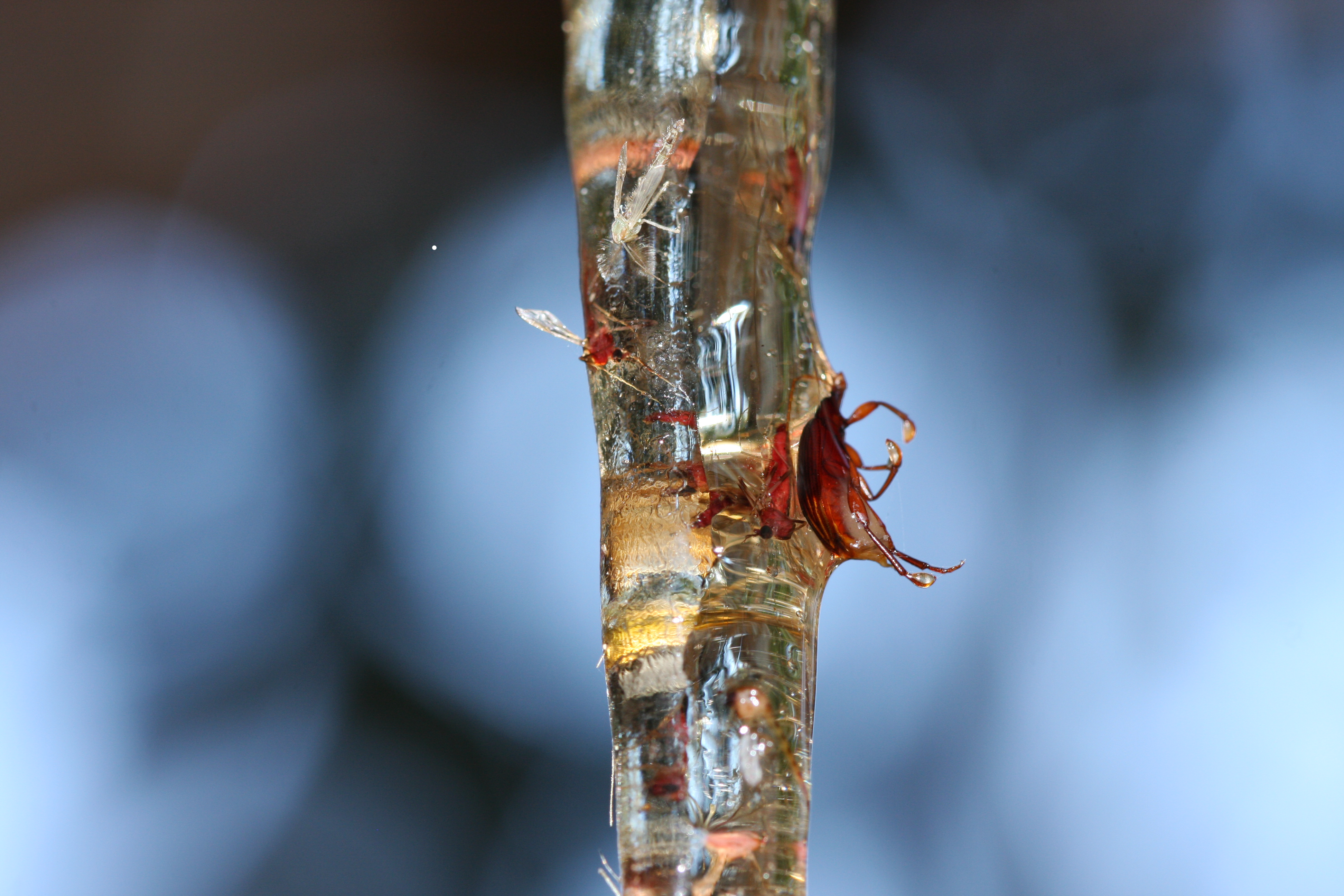

Supplement: S2 Fig — (TIF) [file pone.0232623.s002.tif]
