# Supplementary material for: Unravelling the mystery of “Madagascar copal”: Age, origin and preservation of a Recent resin
Source: PLoS One. 2020 May 18;15(5):e0232623. doi: 10.1371/journal.pone.0232623 (PMC7233546; doi:10.1371/journal.pone.0232623)

PLOS ONE

"Madagascar copal": a new approach to study the loss of biodiversity during the Anthropocene

--Manuscript Draft--

**Manuscript Number:**	PONE-D-19-32304
**Article Type:**	Research Article
**Full Title:**	"Madagascar copal": a new approach to study the loss of biodiversity during the Anthropocene
**Short Title:**	"Madagascar copal" as a container of Anthropocene biodiversity
**Corresponding Author:**	Xavier Delclos, Ph. D.<br>Universitat de Barcelona<br>Barcelona, Barcelona SPAIN
**Keywords:**	Copal; resin; taphonomy; radiocarbon-dating; organic geochemistry, soil deposits; Anthropocene; biodiversity loss; Africa; Madagascar
**Abstract:**	The loss of biodiversity during the Anthropocene is a topic in constant discussion, especially this in the most biodiverse hotspots, such as Madagascar. In this regard, the study of ancient organisms, like those included in "Madagascar copal", is of relevance. The “Madagascar copal" originated from the leguminous tree Hymenaea verrucosa, which produced and produces resin abundantly. In the last 20 years, interest has focused on the scientific study of its biological inclusions, mainly arthropods, described in dozens of publications. The age and origin of the deposits of "Madagascar copal" have not yet been resolved. Our objectives are to determine its geographical origin and age, and thus increase its importance as a source of biological/paleobiological information. Although Hymenaea was established in Madagascar during the Miocene, we did not find geological deposits of copal or amber in the island. It is plausible that the evolution of those deposits was negatively conditioned by the type of soil, by the climate, and by the development of soil/litter microorganisms, which avoid preservation of the resin pieces in the litter and subsoil over 300 years. Our results indicate that "Madagascar copal" is a Recent resin up to a few hundreds of years old and originated by Hymenaea trees that live in the lowland coastal forests, one of the most endangered ecosystems in the world. The included and preserved biota is representative of that ecosystem today or during historical times and have relevant implications for the studies about Anthropocene biodiversity loss in this hottest hotspot.
**Order of Authors:**	Xavier Delclos, Ph. D.
	Enrique Peñalver
	Voajanahary Ranaivosoa
	Mónica Morayma Solorzano Kraemer
**Additional Information:**	
**Question**	**Response**
**Financial Disclosure**<br><br>Enter a financial disclosure statement that describes the sources of funding for the work included in this submission. Review the submission guidelines for detailed requirements. View published research articles from *PLOS ONE* for specific examples.<br><br>This statement is required for submission and **will appear in the published article** if	The funders had no role in study design, data collection and analysis, decision to publish, or preparation of the manuscript.

the submission is accepted. Please make sure it is accurate.<br><br>**Unfunded studies**<br>Enter: *The author(s) received no specific funding for this work.*<br><br>**Funded studies**<br>Enter a statement with the following details:<br>• Initials of the authors who received each award<br>• Grant numbers awarded to each author<br>• The full name of each funder<br>• URL of each funder website<br>• Did the sponsors or funders play any role in the study design, data collection and analysis, decision to publish, or preparation of the manuscript?<br>• **NO** - Include this sentence at the end of your statement: *The funders had no role in study design, data collection and analysis, decision to publish, or preparation of the manuscript.*<br>• **YES** - Specify the role(s) played.<br><br>* typeset	
**Competing Interests**<br><br>Use the instructions below to enter a competing interest statement for this submission. On behalf of all authors, disclose any competing interests that could be perceived to bias this work—acknowledging all financial support and any other relevant financial or non-financial competing interests.<br><br>This statement **will appear in the published article** if the submission is accepted. Please make sure it is accurate. View published research articles from *PLOS ONE* for specific examples.	The authors have declared that no competing interests exist.

Powered by Editorial Manager® and ProduXion Manager® from Aries Systems Corporation

**NO authors have competing interests**<br><br>Enter: *The authors have declared that no competing interests exist.*<br><br>**Authors with competing interests**<br><br>Enter competing interest details beginning with this statement:<br><br>*I have read the journal's policy and the authors of this manuscript have the following competing interests: [insert competing interests here]*<br><br>* typeset	
**Ethics Statement**<br><br>Enter an ethics statement for this submission. This statement is required if the study involved:<br><br>• Human participants<br>• Human specimens or tissue<br>• Vertebrate animals or cephalopods<br>• Vertebrate embryos or tissues<br>• Field research<br><br>Write "N/A" if the submission does not require an ethics statement.<br><br>General guidance is provided below. Consult the submission guidelines for detailed instructions. **Make sure that all information entered here is included in the Methods section of the manuscript.**	N/A

Powered by Editorial Manager® and ProduXion Manager® from Aries Systems Corporation

**Format for specific study types**<br><br>**Human Subject Research (involving human participants and/or tissue)**<br>• Give the name of the institutional review board or ethics committee that approved the study<br>• Include the approval number and/or a statement indicating approval of this research<br>• Indicate the form of consent obtained (written/oral) or the reason that consent was not obtained (e.g. the data were analyzed anonymously)<br><br>**Animal Research (involving vertebrate animals, embryos or tissues)**<br>• Provide the name of the Institutional Animal Care and Use Committee (IACUC) or other relevant ethics board that reviewed the study protocol, and indicate whether they approved this research or granted a formal waiver of ethical approval<br>• Include an approval number if one was obtained<br>• If the study involved *non-human primates*, add *additional details* about animal welfare and steps taken to ameliorate suffering<br>• If anesthesia, euthanasia, or any kind of animal sacrifice is part of the study, include briefly which substances and/or methods were applied<br><br>**Field Research**<br><br>Include the following details if this study involves the collection of plant, animal, or other materials from a natural setting:<br>• Field permit number<br>• Name of the institution or relevant body that granted permission	
**Data Availability**<br><br>Authors are required to make all data underlying the findings described fully available, without restriction, and from the time of publication. PLOS allows rare exceptions to address legal and ethical concerns. See the PLOS Data Policy and FAQ for detailed information.	Yes - all data are fully available without restriction

Powered by Editorial Manager® and ProduXion Manager® from Aries Systems Corporation

A Data Availability Statement describing where the data can be found is required at submission. Your answers to this question constitute the Data Availability Statement and **will be published in the article**, if accepted.<br><br>**Important:** Stating 'data available on request from the author' is not sufficient. If your data are only available upon request, select 'No' for the first question and explain your exceptional situation in the text box.<br><br>Do the authors confirm that all data underlying the findings described in their manuscript are fully available without restriction?	
**Describe where the data may be found in full sentences. If you are copying our sample text, replace any instances of XXX with the appropriate details.**<br><br>• If the data are **held or will be held in a public repository**, include URLs, accession numbers or DOIs. If this information will only be available after acceptance, indicate this by ticking the box below. For example: *All XXX files are available from the XXX database (accession number(s) XXX, XXX.*).<br>• If the data are all contained **within the manuscript and/or Supporting Information files**, enter the following: *All relevant data are within the manuscript and its Supporting Information files.*<br>• If neither of these applies but you are able to provide **details of access elsewhere**, with or without limitations, please do so. For example:<br><br>*Data cannot be shared publicly because of [XXX]. Data are available from the XXX Institutional Data Access / Ethics Committee (contact via XXX) for researchers who meet the criteria for access to confidential data.*<br><br>*The data underlying the results presented in the study are available from (include the name of the third party*	All relevant data are within the manuscript and its Supporting Information files.

and contact information or URL).<br>• This text is appropriate if the data are owned by a third party and authors do not have permission to share the data.<br><br>* typeset	
Additional data availability information:	Tick here if the URLs/accession numbers/DOIs will be available only after acceptance of the manuscript for publication so that we can ensure their inclusion before publication.

Manuscript
Click here to download Manuscript Anthropocene Malagasy copal_final version_PlosOne.pdf

"Madagascar copal": a new approach to study the loss of biodiversity during the Anthropocene

Xavier Delclòs[1*¶], Enrique Peñalver[2¶], Voajanahary Ranaivosoa[3], Mónica M. Solórzano Kraemer[4¶]

[1] Departament Dinàmica de la Terra i de l'Oceà, Facultat de Ciències de la Terra, and Institut de Recerca de la Biodiversitat (IRBio), Universitat de Barcelona, Barcelona, Spain
[2] Instituto Geológico y Minero de España (Museo Geominero), Valencia, Spain
[3] Département Bassins sédimentaires, Evolution et Conservation, Faculté des Sciences, Université d'Antananarivo, Antananarivo, Madagascar
[4] Senckenberg Research Institute, Frankfurt am Main, Germany

*Corresponding author
E-mail address: xdelclos@ub.edu (XD) – permanent address

Abstract

The loss of biodiversity during the Anthropocene is a topic in constant discussion, especially this in the most biodiverse hotspots, such as Madagascar. In this regard, the study of ancient organisms, like those included in "Madagascar copal", is of relevance. The "Madagascar copal" originated from the leguminous tree *Hymenaea verrucosa*, which produced and produces resin abundantly. In the last 20 years, interest has focused on the scientific study of its biological inclusions, mainly arthropods, described in dozens of publications. The age and origin of the deposits of "Madagascar copal" have not yet been resolved. Our objectives are to determine its geographical origin and age, and thus increase its importance as a source of biological/paleobiological information. Although *Hymenaea* was

established in Madagascar during the Miocene, we did not find geological deposits of copal or amber in the island. It is plausible that the evolution of those deposits was negatively conditioned by the type of soil, by the climate, and by the development of soil/litter microorganisms, which avoid preservation of the resin pieces in the litter and subsoil over 300 years. Our results indicate that "Madagascar copal" is a Recent resin up to a few hundreds of years old and originated by *Hymenaea* trees that live in the lowland coastal forests, one of the most endangered ecosystems in the world. The included and preserved biota is representative of that ecosystem today or during historical times and have relevant implications for the studies about Anthropocene biodiversity loss in this hottest hotspot.

Keywords: Copal; resin; taphonomy; radiocarbon-dating; organic geochemistry, soil deposits; Anthropocene; biodiversity loss; Africa; Madagascar

Introduction

Madagascar is an island located in Eastern Africa in the Indian Ocean (Fig 1), between 11°57 S and 25°29 S, considered one of the few hottest hotspots of biodiversity in the world (Myers et al., 2000), especially in relation to the tropical evergreen rainforest, which extends parallel to the east margin of the island. For more than 80 million years, Madagascar has been isolated from the Africa continent, which is why paleobiological studies of the biota are of great relevance (Krause et al., 2019). Nevertheless, it is postulated that a land bridge connected Africa and Madagascar between 45 and 26 Ma ago (McCall, 1997). During the Miocene the continent of Africa was covered extensively by tropical rainforests (Burgoine et al., 2005), however, the presence of paleosoils and dry fossil-floras seem to contradict in part this idea (Retallack et al., 2002).

Fig 1. Map showing the countries that surround the Mozambique Channel, with the localities cited in the text.

The biota embedded in resins, sub-fossil resins, and ambers are preserved three-dimensionally with external and some internal characters in fine detail, sometimes soft-parts included, providing one of the best-preserved arthropod assemblages and valuable paleobiological data. The study of inclusions in copal has been argued as important for the knowledge of the diversity that could be extinct or undiscovered (Solórzano Kraemer et al., 2015; Penney, 2016). Copal usually contains extant, undescribed species, so they can scarcely increase knowledge of the evolution of insects. However, it may be crucial for the detection of changes during the Anthropocene in resin-producing forest ecosystems, principally considering the current rapid, human induced, modification of the terrestrial environments.

The age of the “Madagascar copal” has not been accurately studied, mainly because of problems to find and to study the geology of possible outcrops. However, in the last twenty years, several articles on arthropods included in the "Madagascar copal" have been published (i.e., Lourenço, 1996; Vitali, 2007; Bosselaers et al., 2010; Bukejs and Legalov, 2017). For all of them the age and the geographical origin of the studied samples were imprecise or unavailable. The age of the “Madagascar copal” has been attributed to a broad age range from tens of years to a few million years (i.e., Poinar, 1992, 1999; Labandeira, 2014).

The research carried out in Madagascar by us during the last six years focused on the leguminous tree *Hymenaea verrucosa* Gaertner, 1791 (Solórzano Kraemer et al., 2018) invited us to answer two questions that we consider essential for the development of biological and paleobiological investigation of bioinclusions contained in resins: 1) How old is the so called "Madagascar copal" from which a large list of taxonomic works on its bioinclusions have been published, and 2) what is its geographical origin?

In paleontological and biological studies, the precise location of the collection-site and its dating ~~is~~ mandatory. From this point of view, it is especially worrisome the amount of published works concerning the biota included in the "Madagascar copal” lacking these data with consequences for our knowledge of the past. Some species studied within the "Madagascar copal" have been previously found today in nature (i.e., Lourenço, 2000; Vitali, 2006; Dikow and Bayless, 2009; Bosselaers et al., 2010), but others are only known from copal (i.e., Guilbert and Heiis, 2016; Bukejs and Legalov, 2017). Our study indicates that the nature of the “Madagascar copal” has long been misinterpreted and better explains the presence of Recent species inside it.

Historical background

Copal as scientific term

The scientific term “copal” is a problem, mainly because it is used without considering any typology of resins based on their different states of polymerization and is used in both commercial and scientific fields, such as geology, geochemistry or paleontology, creating great confusion. Copal is considered an intermediate step of formation between resin and amber, or tree resin that is not completely fossilized (polymerized), but ~~for that process~~ a specific time is not assigned. The term copal is derived from the word *copalli* (in Aztec *náhuatl*) ~~that means~~ resin used as incense. Major Pre-Hispanic cultures used it to refer to resins produced by different tree genera such as *Protium* Burman, 1768 and *Bursera* von Jacquin ex Linnaeus, 1762 (Burseraceae), *Pinus* Linnaeus, 1753 (Pinaceae) and *Liquidambar* Linnaeus, 1753 (Altingiaceae). The Spanish people established in the Americas during the 15th –16th centuries

applied the term copal also for resins of different trees used by the Aztecs (Langenheim, 2003), and later until today the term was broadly used for all non-fossilized resins worldwide.

The classification of resin or amber is based on the ensemble of chemical compounds contained (Anderson and Crelling, 1995). The classification can be more direct if the amber or resin can be associated to a living or fossil tree. Neither copal nor amber have a crystalline structure, but as a tree product both are organic materials. Polymerization of organic hydrocarbon molecules changes the resin to sub-fossil resin, and a cross-linkage reaction between chains of these hydrocarbons produces amber (Clifford and Hatcher, 1995). Copal and amber can be differentiated by FTIR spectroscopy by observing precise exocyclic methylene bands (Grimalt et al., 1988).

The age of the sedimentary deposit is a criterion used to consider a subsoil resin as copal and is often limited between 10,000 years and 5 million years (i.e., Poinar, 1992; Kosmowska-Ceranowicz, 2006). Schlüter and Glöckner (1978) considered that a one million years old resin is to be called amber, while when it is younger, copal. Anderson (1996, p. 252), based on objective analyses, proposed a scale based on ^{14}C dating according to "*fresh resins and ambers are members of a continuous series*". For Anderson, resin from less than 250 years old should be named "modern resin", "ancient resin" when it was between 250 and 5,000 years old, and "sub-fossil resin" for an age between 5,000 and 40,000 years; he did not consider using the term copal for any of the temporal terms of development from resin to amber. Langenheim (2003, p. 146) considered the term "copal" controversial but generated confusion when she ~~noted his~~ intention to follow the terminology proposed by Anderson (op. cit.), and in later chapters used the expression "hard copals" without indication of Anderson's terminology (pp. 392). She even used again the term "copal" to refer to the resin found in the fruit pods of *H. verrucosa* (pp. 397). Kimura et al. (2006 a, b) considered that the polymerization that transforms copal into amber proceeds very slowly. They proposed that it takes about 17 million years to reach the halfway

point; this means that copal requires some millions of years to mature into amber. Vávra (2009, p. 215) considered copal as a "*Resins having not yet undergone all the steps of fossilization like polymerisation and maturation*". Lambert et al. (2012) considered that the resin of thousands of years to a million years old better retains its original molecular structure and is better termed "copal".

Thermal analysis allows an estimation of the age of resins and ambers (Feist et al., 2007). In this way "Madagascar copal" has been dated from Holocene to Recent (1,000–100 y) by Ragazzi et al. (2003). However, until today there is no technique using the loss of volatile components as an age indicator for fossil or sub-fossil resins, this is probably because of the complexity of these components (McCoy et al., 2017). It must be remembered that there is a gap of 13 to 15 million years between the youngest amber deposits dating from the Middle Miocene (i.e., New Zealand, Peruvian, Mexican, and Dominican amber) and the oldest copal deposits, which are a few thousand years old (i.e., New Zealand, Colombian or Dominican copals).

Copal as a trade product in East Africa

Madagascar houses several angiosperm resin-producing trees, mainly in the oriental area of the island in the evergreen rainforests such as *Canarium* spp. (Burseraceae), and in the lowland coastal forest such as *Hymenaea verrucosa* (Leguminoseae) (Fig 2). The resin produced by *Canarium* spp. was historically called by merchants as "elemi" or "alemi", and "copal" was used for the resin produced by *H. verrucosa* in the context of the Easter African trade (Regert et al., 2008).

Fig 2. *Hymenaea verrucosa*. Madagascar: (A) Trunk, in Sacaramy (Antsiranana) with an important injury in its base. (B) Habitus of an individual, producing abundant resin in several parts, Andranotsara (Sambava). C) Detail of the injury in A, with important resin production. (D) Leaves, Ambahy (Mananjary) (leaflet length ca. 6.5 cm). (E) Decomposed fruit showing seeds and pod resin beads (these developed as resin pockets in the pod wall) and intact fruit 4 cm in

length, Ambahy (Mananjary). Observe the characteristic roughness of the external surface produced by these resin pockets, and which gives the name to the species.

"East African copal" was exported almost since 2,500 BCE (i.e., Meyer et al., 1991; Horton, 1996; Sunseri, 2007; Pollard and Kinyera, 2017). It has been commonly found in archaeological sites from East Africa since the Early Iron Age (2nd–3rd centuries BCE, Chami, 2003; Crowther et al., 2015), and intensely traded from the 11th century (i.e., Horton, 1996; Regert et al., 2008) to the beginning of the 20th century from Eastern Africa to diverse countries. This resin was traded for thousands of years together with other resinous products such as gum arabic (Fabaceae: *Acacia* Miller, 1754), myrrh (Burseraceae: *Commiphora* von Jacquin, 1797) and frankincense (Burseraceae: *Boswellia* Roxburgh ex Colebrooke, 1807) (Meyer et al., 1991; Regert et al., 2008). All these resins or oleoresins and copal can be differentiated by their diterpenic or triterpenic geochemical characters (Daher et al., 2013a). "East African copal" is a diterpenic resin containing ozic acid, an enantiomer of communic acid that polymerizes as soon as it is exuded from the tree in contact with air and light (Cunningham et al., 1977).

Meyer et al. (1991) found a pendant made with "copal" of *Hymenaea verrucosa* dated from 2,500 to 2,400 BCE in an Iraqi grave, the oldest pieces of copal with bioinclusions from East Africa are dated from 2,000 BCE and have been found in Denderah (Egypt), where more than 30% of the "copal" pieces (mainly manufactured beads) found have bioinclusions (Creamer, 2014).

The "copal" obtained in countries on both sides of the Mozambique Channel (mainly Tanzania, Mozambique and Madagascar) has been regularly traded as "East African copal" or "Zanzibar copal". This was because the Sultanate of Zanzibar (today Tanzania) was for centuries the central hub of the mercantile activities between Africa and the other harbors of the Indian Ocean, such as the Malagasy harbors (Sanchez, 2015). By the 16th century, Zanzibar began to

trade "copal" with Europe under the denomination "gum copal" or "Zanzibar copal". During the 17th century, the whole trade was managed and controlled from Zanzibar. Although it received the name of "Zanzibar copal", the Zanzibar Island only yielded small quantities and of lower quality of this commercial product. While Zanzibar was the commercial center during the 17th century, Madagascar seems not to have been integrally locked into the Western Indian Ocean commercial network until the beginning of the 18th century. Madagascar centered all its commerce in Mahajanga and the other Malagasy harbors of the west coast, since the eastern coast of Madagascar was dominated by the Merina Empire (Campbell, 1993).

While trade between Madagascar and the east coast of Africa possibly began in the 7th century, there is no documentation that the "Madagascar copal" trade began before the mid-18th century (Beaujard, 2007). Possibly the first reference to "Madagascar copal" as an exportable merchandise was in 1767 when Valigny (in Blanchard et al., 1902) reported a Malagasy resin which he believed to be that called "*sandrousse*" by the Chinese. Ten years later Benyowski (in Blanchard et al., 1902) wrote about the gum "*sanderousse*" as derived from the African name "*sandarusi*". At the end of the 18th century, the "copal" was exported in large quantities from the harbors of the east coast of Madagascar (Meldola, 1891) to Europe, the United States of America and some Southeast Asian countries such as India and China, to be used in the manufacture of varnishes. Around 1863, 300 tons of "copal" were collected annually in Madagascar, while between 1868 and 1880 the annual exportation of "Madagascar copal" fluctuated between nothing and 80 tons (Blanchard et al., 1902). Nevertheless, the trade of "copal" in Madagascar was not developed extensively, unlike in Tanzania, mainly because the Malagasy people did not take great care in its preparation, in cleaning its impurities, which considerably reduced the price (Maundrell, 1867).

"Madagascar copal", geographical origin and age

Elton (1874) and McIntosh (1908) described the extraction of the "Zanzibar copal" in Tanganyika from the subsoil, below 120 cm deep, and, indeed, very few diggers appear to go beyond 90 cm deep. According to Rice (2006), the “Zanzibar copal” was found by natives from about 60 cm to 120 cm underground over a wide area on the coast opposite the island of Zanzibar.

The first sure reference of “Madagascar copal” as a trade name was from Filhol (1842–43). For Filhol ~~the~~ “Madagascar copal” was hard copal traded as large flat, yellow pieces to India. ~~The~~ “Madagascar copal” was collected from the soil and, according to Howes (1949, p. 98), “*Madagascar copal resembles that from East Africa in hardness and other characters but does not show the characteristic "goosekin". It is collected from the soil and may be present in large deposits mainly in the north part of the island. This copal is considered to be derived from the same botanical source as the East African. Resin may also be obtained by tapping (De Cordemoy)*”. In 1868, Oliver said (p. 71) that “*Copal gum might be collected in Madagascar to almost any extent…there was but little collected by the natives*”. During the second half of the 19th century, officers and soldiers of garrisons located on the Malagasy coast forced the people of the surrounding towns to deliver part of their products at low prices, which they resold to the merchants, or sent their slaves to collect both bee wax and “copal” (Jacob, 1996). Nevertheless, the “copal” harvested was only an accessory to ~~that of~~ rubber collection and trade (McIntosh, 1908); the “copal” was not sufficiently remunerative to harvesters, but it served to compensate the loads they brought to the coast. The whole of this trade was~~, moreover,~~ controlled by Indians and Arabs established at Vohemar and Antsiranana. At the end of the 19th century “copal” was also harvested and sold as merchandise in the region of Tamatave (Gallieni, 1908).

All these ancient references suggest that "Madagascar copal" was obtained in the north and the east coastal regions of Madagascar (Fig 1), where today the resin-producing tree *Hymenaea verrucosa* lives (Fig 2). Also, Rakotovao (2015) proposed the area of Antsirarana as the source of the "copal" and dated it as Quaternary or Holocene. Previous authors (i.e., Kirk, 1876; Langenheim and Lee, 1974; Langenheim, 1995) indicated the species *H. verrucosa* as the producer tree of the "Madagascar copal". However, comparisons with other large resin producers in Madagascar, such as *Canarium* Linnaeus, 1759 (Bursaceae), very abundant in the evergreen rainy forests (such as *Montagne d'Ambre,* Masoala or Marojejy in Sambava*)*, have not been made.

The presence of *Hymenaea* in Madagascar needs to be explained. Several studies on geology and biota phylogenies (i.e., Crottini et al., 2012; Bruneau et al., 2014; Federman et al., 2015; Klimke et al., 2017) strongly suggest that the genus is too young to explain its presence in Madagascar by vicariance. Phylogenetic analysis by Fougère-Danezan (2006) considered that *Hymenaea* started in continental Africa, as previously suggested by Langenheim and Lee (1974), along with its sister genus *Guibourtia* Bennett, 1857 which lives today in Western African forests. Fougère-Danezan (2006) suggested that the clade [*Hymenaea* + *Guibourtia*] originated in Africa 25 to 19 Ma ago. This has been corroborated by the discovery of the "amber of Ethiopia", initially dated from the Cenomanian (Schmidt et al., 2010), and after re-evaluation by Perrichot et al. (2016, 2018) as Miocene and produced by the genus *Hymenaea* (McCoy et al., 2017). Later, the uplift of the East African Plateau during the Miocene, about 13.5 Ma ago (Wichura et al., 2011), favored the development of *Guibourtia* in the occidental margin with more humid environments, and *Hymenaea* in the oriental lowlands with better adaptation to annual periods of water scarcity. The East African Plateau limited the *Hymenaea* expansion westward (Burgess et al., 1998) and favored its dispersion to several Indian Ocean islands.

Zoochory is not reported as dispersal mechanism for *H. verrucosa* in East Africa and Madagascar, nevertheless, the fauna, mainly rodents and other mammals, can disperse and transport fruits and seeds of Neotropical *Hymenaea* species (Lee and Langenheim, 1975). Hydrochory is the main mechanism for seed dispersal of the buoyant fruits. Thus, dispersion of *Hymenaea* possibly occurred via short distance dispersal across Mozambique Channel by birds and/or marine currents. However, marine currents during the Upper Miocene till today were and are largely unfavorable for marine dispersal from Africa to Madagascar (i.e., Ali and Huber, 2010; Samonds et al., 2012; Peris et al., 2015). Madagascar is surrounded by several important oceanic currents (Lutjeharms et al., 1981; Hawlitschek et al., 2016). The present-day surface currents in the Mozambique Channel would not have facilitated west to east transoceanic dispersion by floating seed dispersal. *Hymenaea verrucosa* possibly disseminated from East Africa to Madagascar and surrounding islands during the Early Miocene, mainly during hurricane ~~periods~~ (Ali and Hubber, 2010; Springer et al., 2011). This species is restricted to lowland coastal forests; normally water-dispersed seeds germinate near the water down which they have been dispersed. Long distance dispersion of *Hymenaea* from Southeast Asia via the South Equatorial Current appears not to be plausible, since the genus has never been found in wild Southern Asia forests or as fossil occurrences (see Kew Science, 2019), although it has recently been introduced by humans.

Because the presence of *Hymenaea* in Madagascar goes back to the Miocene, it would conceivable to find deposits, not only of copal but also of amber, originated from resin of this resin-producing tree. However, Meunier (1909, 1916), based on the study of its inclusions, had doubts about the old age of East African copal; also, Williamson (1932) considered the "Madagascar copal" of Recent origin. Hills (1957) already suggested that the study of bioinclusions permits the differentiation of amber from copal and considered that amber includes mainly extinct insect species, while copal includes mainly Recent insect species. Nevertheless,

he does not differentiate between copal and Recent resin. According to Vankerkhoven et al. (2010) the age of the "Madagascar copal" is uncertain and varies with the depth of mining, but the fauna included in the copal pieces differs from the Recent fauna, which suggests that at least a part of it was formed several thousand years ago. Vankerkhoven's statement is not correct because: i) there is no evidence that Malagasy people obtained the "Madagascar copal" from ~~the~~ underground, ii) studied pieces do not have a proper geographic register, and iii) Recent arthropod fauna is so far inadequately known in the hotspot Madagascar. Poinar (1992) considered the existence of copal in Madagascar attributing an age between 3 and 4 Ma, while Le Gall et al. (2010) ~~gave to it~~ an age between 1 and 2.6 Ma, in both cases without evidence. Other authors such as Schlüter and von Gnielinski (1987), Lambert et al. (1995), Poinar (1999), Vávra (2009) or Stach et al. (2019) suggested that the "Madagascar copal" from the north coast (Antsiranana) had a presumed age from 10,000 to 50,000 years-old; in no case was the exact provenance of the analyzed pieces indicated.

Material and methods

As a new approach to determine the actual age we have proceeded to make two systematic pits and subsequent radiocarbon dating analyses (^{14}C) of the resin samples obtained at different depths in two different *Hymenaea* forests, as well as of some pieces housed in museums and others obtained in local markets. To know the potential localities from where ~~the~~ "Madagascar copal" was obtained, either by excavation in the soil or maybe by harvesting fresh resin from the *H. verrucosa* trees~~,~~ first an intense bibliographical search was carried out that allowed us to delimit three main geographic regions; these are Mananjary in the southeast, *Montagne d'Ambre* - Antsiranana in the north, and Sambava on the northeast (Fig 1). In these regions, taphonomic and geological studies have been carried out in search for copal-bearing geological deposits.

Collection localities and environmental characteristics

The search for copal deposits (during ~~years~~ 2013, 2015, and 2017), was confined to the three areas that have traditionally been proposed in the literature as the origin of the copal used in taxonomic studies (Fig 1).

1. *Mananjary region* (2013, research area between Nosy Varika and Ambahy). *Hymenaea* is common and lives here under an equatorial climate (Af in the Köppen-Geiger climate classification system, see Kottek et al., 2006). The average annual temperature is 23.1 °C (the variation in annual temperature is around 6.2 °C). The annual rainfall is high, average 2,439 mm, with precipitation even during the driest month. We harvested resin directly from the *H. verrucosa* trees (Solórzano Kraemer et al., 2018). *Hymenaea* trees are used for shadow to the vanilla plants.

2. *Montagne d'Ambre - Antsiranana region* (2015, research area between Sacaramy and Antsiranana). *Montagne d'Ambre* (Amber Mountain) is usually reported by Malagasy dealers and in some publications (i.e., Lourenço, 2000; Poinar et al., 2001) as the locality where "Madagascar copal" is found. There, people do not know about the origin of the names *Montagne d'Ambre* or *Cap d'Ambre*, but in maps from the 16th century (1588, Africae Nova Tabula), *C. del Ambar* (Amber Cape) is a location more to the north of Madagascar. *Montagne d'Ambre* corresponds to an evergreen rainy forest that is not conducive to the development of the *Hymenaea* forests. Between Antsiranana (= Diego Suarez, close to "*Cap d'Ambre*"), and Sacaramy, *Hymenaea* lives under a tropical savanna climate (Aw in Köppen-Geiger classification). Compared to winter, the summer is much rainier. The average annual temperature is 26.5 °C; the temperature in December averages 28.0 °C., at 24.8 °C on average, July is the

coldest month. The average annual rainfall is 1,156 mm. The driest month is September, with 11 mm of rain. In January, the precipitation reaches its annual peak, with an average of 320 mm.

3. *Sambava region* (2017, research area between Andranotsara and Antampolo). In this region the genus lives under an equatorial climate (Af in Köppen-Geiger classification). The rainfall in Sambava is significant, with precipitation even during the driest month. The temperature averages 24.7 °C; the warmest month is February, with an average temperature of 26.8 °C.; at 22.2 °C on average, August is the coldest month. The annual average rainfall is 2,030 mm; the driest month is November, with 77 mm of rainfall; in January, the precipitation reaches its annual peak, with an average of 257 mm. In this region the trees are also used to shadow the vanilla plants, to obtain stems for coal production, and branches and trunks to build. In this area two pits to study the resin accumulation and burial were dug as described below. To recognize the soil horizons of the Malagasy *Hymenaea* forests and the possible preservation/degradation of resin in it, two test pits were dug, 150 x 150 cm ~~of~~ area (Fig 4). One was dug in Andranotsara (pit Q1), ~~of~~ 175 cm deep (GPS: S14º 38.472’ - E050º12.467’) (Figs 4A–B) and a second in Antampolo (pit Q2), 140 cm deep (GPS: S14º 43.468’ - E050º12.860’) (Fig 4C). The grid was made with nails and rope and excavated with picks and shovels to obtain copal or resin samples from different levels in depth. Two synthetic pedologic logs (Figs 5A–B) were drawn (description below, section 4.2), and soil samples were collected from each of the differentiated horizons and sub-horizons (Figs 4D–G, 8A–B, 8E–G).

FTIR analyses

In the Scientific Facilities of the University of Barcelona we conducted Fourier transform infrared spectroscopy (FTIR) analyses of sub-fossil/Recent resins and ambers. We used a diamond PerkinElmer FT-IR Spectrometer Frontier; software version 10.4.2 (2014). Analyzed

samples are housed at the University of Barcelona. Samples studied are the following: 1) one sample of resin from *Canarium madagascariensis* Engler, 1883 collected in 2013 in the Ranamofana National Park, Madagascar, piece number RMCR-0[illegible]) two samples of “Madagascar copal” from the Antsirabé market, piece number CM01 containing a *H. verrucosa* flower and 3) piece number CM02 containing a *H. verrucosa* leaflet, 4) one sample of resin, piece number RHvA01_R1 from a *H. verrucosa* branch collected in Ambahy, Mananjary in 2013, 5) sample of resin from *H. verrucosa* of the Andranotsara pit Q1 from A horizon (0–10 cm depth), piece number CM05_Q1, and 6) sample of resin from B horizon (10–20 cm depth), piece number CM06_Q1, 7) sample of resin from *H. verrucosa* of the Antampolo pit Q2 from A Horizon (3–10 cm depth), piece number CM03_Q2, and 8) sample of resin from B horizon (10–20 cm depth), piece number CM04_Q2, 9) one sample of resin from “copal of Colombia” from the Santander region, piece number CCS01, which includes a lot of winged termites, possibly originating from *H. courbaril*, 10) one sample of Mexican amber (Lower to Middle Miocene), from Simojovel de Allende, piece number AMS01, 11) two different pieces of Dominican amber (Middle Miocene) from El Valle region pieces number AD-03 (Siete Cañadas) and 12) AD-04 (El Cabao-San Rafael); Mexican and Dominican ambers were produced by three different *Hymenaea* spp., and 13) one sample of Ethiopian amber (Middle Miocene) produced by *Hymenaea* sp., piece number EA-01, piece housed in the Naturmuseum Senckenberg (Frankfurt). To characterize the original material of the soil beads found abundantly in the pit horizons O and A of the pits dug, FTIR analyses have also been carried out (Fig 5C); 14) sample of resin of living *H. verrucosa* tree from Antampolo, piece number RHvA, 15) sample of several soil beads found in the O horizon of the pit Q2 soil, and 16) sample of several soil beads found in the surface of the soil in another place of Antampolo. This variety of samples has been analyzed to permit comparison between them (Figs 3A–C),

Radiocarbon-dating of the "Madagascar copal"

The ^{14}C analyses were done by Beta Analytic laboratories, USA, who reported the age results in "pMC" (percent modern carbon) units rather than BP. Beta Analytic reports in the pMC format when the analyzed material has more ^{14}C than did the modern (AD 1950) reference standard. We analyzed 5 pieces of "Madagascar copal": 1) piece acquired in the market of Ansirabé (piece number CM01); seller said that the origin was *Montagne d'Ambre,* but most likely it came from the area of Mananjary; 2) piece of "copal" from Andranotsara (Sambava region) with precise information about its origin, collected at about 50 cm deep from the soil, close to the Andranotsara Pit Q1 (piece number SMF Be 13311); 3) piece of "copal" from the Senckenberg Research Institute (Frankfurt am Main, SMF) collection, SMF Be 3724, which was acquired between the years 1874 and 1901 exhibiting a label indicating "Zanzibar copal"; 4) piece from the Andranotsara pit Q1, being one of the deeper resin/copal pieces found in it, at 50 cm (Fig 8G); and 5) piece from the Antampolo pit Q2, being one of the deeper resin/copal pieces found in it, at 25 cm (Fig 8F). A portion of each these pieces are housed in the University of Barcelona (Faculty of Earth Sciences).

SEM imaging

The micromorphology of the sectioned soil beads used a FEI INSPECT (5350 NE Dawson Creek Drive Hillsboro, Oregon 97124, USA) Scanning Electron Microscope (SEM) at the Museo Nacional de Ciencias Naturales, CSIC (Madrid).

Results and discussion

Resiniferous tree that originated the "Madagascar copal"

The FTIR analyses carried out on resin, copal and amber from different areas, but geographically well located (see section 3.2) confirm that the "Madagascar copal" was produced by the fabacean species *Hymenaea verrucosa.* The resiniferous tree species *Canarium madagascariensis* inhabits the *Montagne d'Ambre*, however no copal ~~was~~ originated from the genus *Canarium* (Fig 3A). Copal and amber can be differentiated by FTIR spectroscopy by observing the exocyclic methylene bands at 887, 1642 and 3048 cm^{-1} (Grimalt et al., 1988). In the case of copal, the two last bands are weak, but they can be observed, and the 887 cm^{-1} band is very intense. In the case of ambers these bands are weak or absent (Guiliano et al., 2007). The “Madagascar copal” is characterized by the 1700 > 1730 and 1600 cm^{-1} intense bands which correspond to the Ester and Carboxyl groups, and to the Ester group and >C = C < respectively, and also is characterized by the absence of the 1085 cm^{-1} band (Alcohol group), 972 cm^{-1} band (Ester group), and 792 cm^{-1} band (Aromatic group) (Drzewicz et al., 2016) (Fig 3B). According to McCoy et al. (2017), “Madagascar copal” can be differentiated from other resins or copals from neotropical *Hymenaea* ssp. due to the presence of four organic compounds: 1) 13-epimanool, 2) Caryophyllene, 3) α-curcumene, and 4) Biformene; the first three usually play a role in defense against herbivores, the last one is still unstudied.

Fig 3. FTIR analyses comparing the differences between resin, copal and amber produced by *Hymenaea* spp. and *Canarium madagascariensis*. (A) FTIR analyses of the samples in order to identify the tree-resin producer of “Madagascar copal”: 1. *Canarium madagascariensis* from Ranamofana National Park, Madagascar (RMCR-01); 2–3. two samples of “Madagascar copal” bought in the Antsirabe minerals market (CM01 and CM02); 4–5. two samples of resin from *H. verrucosa* branches of two different trees, in Ambahy (Nosy Varika, Mananjary) (RHvA01_R1 and RHvA02_R0). (B) Malagasy *H. verrucosa* analyses: FTIR analyses that show the comparison between the results in A with other resin, copal and amber produced by *Hymenaea* ssp. The analyses include samples of 1. *Canarium madagascariensis* from Ranamofana National Park, Madagascar (RMCR-01); 2. “Madagascar copal” bought in the Antsirabe minerals market (CM01); 3. resin from *H. verrucosa* branch, in Ambahy (Nosy Varika, Mananjary) (RHvA01_R1); 4. resin from *H. verrucosa* of the Andranotsara pit Q1, found in A horizon A (0–10 cm depth) (CM05_Q1) and 5. in the sub-horizon B_1 (10–20 cm depth) (CM06_Q1); 6. resin pieces of *H. verrucosa* of the Antampolo pit Q2 found in A horizon (3–10 cm depth) (CM03_Q2) and 7. in sub-horizon B_1 (10–20 cm depth) (CM4_Q2). (C)

Neotropical *Hymenaea* spp. resin and amber analyses: 1. resin from “copal of Colombia”, Santander region, possibly from *H. courbaril* (CCS01); 2. Mexican amber from Simojovel de Allende (AMS01); 3. Dominican amber from Siete Cañadas (El Valle) (AD-03) and 4. El Cabao-San Rafael (AD-04) mines, and 5. Miocene amber of Ethiopia (EA-01). Diterpenic resin/copal has some characteristic vibrational group frequencies: characteristic is a low intensity of absorption band at 3080 cm^{-1} that is absent from triterpenoid resin/copal and that corresponds to v(=C-H), intensity absorption band at 2937–2929 cm^{-1} corresponds to v_{as}(C-H), CH_3, CH_2 (methylene group), intensity band at 2874–2844 cm^{-1} corresponds to v_s (C-H), CH_3, CH_2 (methyl group), intensity bands at 1718 cm^{-1}, 1694 cm^{-1}, and 1644 cm^{-1} correspond to v (C=O), intensity band at 1446 cm^{-1} corresponds to δ_{as} (CH_3), intensity band at 1386 cm^{-1} corresponds to δ_s (CH_3), and intensity band at 888 cm^{-1} corresponds out of plane δ (CH_2) of the exomethylene functionality C_8-C_{20}. “Madagascar copal” and “East African Copal” can be differentiated from “Western African Copal” by the linear slope of the spectra in the case of the resin/copal of the West African and the intensity of 3411–3422 cm^{-1} that corresponds to v (OH) of the East African ones. It is possible to differentiate between amber and copal by observing the exocyclic methylene bands at 3048, 1642 and 887 cm^{-1}. In the case of copal, the first two bands are not intense, but they are clearly observed, and the band of 887 cm^{-1} is very intense. In the case of ambers, the bands are absent or with very weak intensities.

The “Zanzibar copal” was related for the first time to the tree species *Hymenaea verrucosa* by Kirk (1876), suggesting that most of the plant remains would belong to the tree that produced the copal, as demo[illegible]ate later by Peñalver et al. (2018). In some samples housed in the Kew Museum of Zanzibar, Kirk observed diverse copal samples containing inclusions of leaves and flowers of *Trachylobium hornemannianum*. Kirk did not know at that time that *T. hornemannianum* had been synonymized a few years earlier with *T. verrucosum* by Oliver (1871). In 1974 the genus *Trachylobium* was considered by Langenheim and Lee as a junior synonym of the genus *Hymenaea, Hymenaea verrucosa* Gaertner, 1791.

The genus *Hymenaea* is a leguminose, belonging to the tribe Detarieae within the subfamily Caesalpinioideae with its origin in continental Africa (Langenheim and Lee, 1974; Fougère-Danezan, 2006). The species *H. verrucosa* lives today in the lowland coastal forest of Kenya, Somalia, Tanzania and Mozambique, mainly between Delgado Cape and Ponta Gomeni near Maputo (Cordemoy, 1911), and in the islands of Madagascar, Zanzibar, and Mauritius (Schlüter and Von Gnielinski, 1986; Langenheim, 2003). It is the unique species of the genus living in Africa and is considered sister of the 14-other species with Neotropical distribution

(Lee and Langenheim, 1975; White, 1983; Langenheim, 2003; Fougère-Danezan et al., 2010), where they were involved in the origin of important Miocene amber and copal deposits (i.e., Iturralde-Vinent, 2001; Solórzano Kraemer, 2007; McCoy et al., 2017). Resin production occurs abundantly within the tribe Detariae that includes more than half of the Caesalpinioideae species (Langenheim, 1995; Fougère-Danezan, 2006). Several Detariae tree genera, such as *Copaifera*, *Daniellia,* and *Hymenaea*, produced the so-called “African copal”, but only the last one lived in ancient Madagascar; neither fossil remains of *Copaifera* nor *Daniellia* are recorded in the island.

Coastal forests are the ideal environment for the development of legumes such as *Hymenaea* due to the occurrence of dry seasonal periods. The high nitrogen metabolism of legumes confers a competitive edge in colonizing seasonally dry environments because leaves can be produced/reduced at convenience under unpredictable climates (McKey, 1994; Schrire et al., 2005). Coastal forests are characterized by distinctive biota because of their development under a climate with a dry season of several months (Burgess et al., 1998; Clarke, 2000; Chidumayo and Gumbo, 2010). This situation conditioned the type of biota that can be found preserved in resin or copal (Solórzano Kraemer et al., 2018), not consisting of evergreen rain tropical forest biota but from coastal or lowland/coastal forest biota. These coastal forests are common in eastern Madagascar; plants are usually rooted in sand bordering lagoons and marshes and usually exposed to salty air washing and hurricane injuries. The coastal forest harbors a very unusual arthropod community (De Gouvenain and Silander, 2003a; Tantely et al., 2016; Gustaf son et al., 2017; Massa, 2017).

Hymenaea verrucosa lives in Madagascar under several climatic regions (sensu Köppen climatic classification): in the southeast (Mananjary region) under an equatorial climate, in the northeast (Sambava region) under a monsoon climate, and in the north (Antsiranana region) under tropical savanna climate. All these areas are included in one of the three Malagasy

ecosystems most endangered. From 2001 to 2017, Madagascar lost 3.27Mha of tree cover, equivalent to a 19% decrease since 2000 (Global Forest Watch, 2019).The east coast littoral forest has shrunk by over 90% of its former distribution and belongs now to the most threatened ecosystems of the world (Ganzhorn et al., 2001; De Gouvenain and Silander, 2003b).

Pit excavations and types of soil

During our collection trips from 2013 to 2017 in the southeast, north and northeast areas of Madagascar, copal deposits were intensively sought (see section 3.1 collection localities for more information). Surprisingly, buried resin pieces were only found in the underground of extant *Hymenaea* forests.

Ninety percent of the total Madagascar land surface is constituted by infertile soils, mainly lateritic clays, lateritic sediments and rockies (Riquier, 1966), what has led it to be called Great Red Island. Lateritic soils are the result of the weathering of very diverse rocks (metamorphic, igneous and sedimentary) and most eastern soils are ferrasols (Ramifehiarivo et al., 2017). The geomorphology of Madagascar roughly consists of three parallel longitudinal zones: 1) the low highland and plains in the west, 2) the central highland, and 3) the humid coastal strip in the east, where *Hymenaea verrucosa* lives today, mainly on salty–sandy soils (regosols *s.l.*). Depending on the areas, regosols have different thicknesses, however *Hymenaea* roots at a shallow depth (1 m maximum; Figs 4B–C)and even large trees are unrooted during the hurricane season.

Oldeman (1990) and Jones et al. (2013) soil classifications include the eastern areas herein studied in the dystric regosols-type (Rd, in FAO Soil Units; RGdy *sensu* Jones et al., 2013), and in the rhodic ferrasols-type the soils of the north area. In ancient French classification of the ORSTOM these soils were described as podzols or pseudo-podzols (Hervieu, 1967; Roederer, 1971). The eastern studied area's rainfall exceeds a mean of 1,500 mm a year, and temperature

averages 24–28ºC with a growing period of more than 270 days. These characteristics are not usual for the formation of regosols because they develop better in arid and semi-arid areas and in mountain regions.

During 2017, two pits were excavated in Sambava region (Fig 4), both in primary *Hymenaea* forest, but partially degraded; one in Andranotsara (Fig 4A-4B) at 101 m to the coast and at 5 m above sea level (Andranotsara pit Q1); and another in Antampolo (Fig 4C) at 1.15 km to the coast and at 18 m above sea level (Antampolo pit Q2). Both were excavated in sandy soils (Ø > 0.063 mm) with slight signs of soil development. They showed very poor cohesion and structure coupled with low water retention capacity and scarce levels rich in organic matter. Based on historical documents, and together with the observations that *H. verrucosa* has superficial roots but of great extension, we hypothesized that the rooted soil quickly loses its organic matter.

Fig 4. Pits excavated in the Sambava region. (A) Commencement of the Andranotsara pit Q1 (S14º38.472' - E050º12.467'). (B) Andranotsara pit Q1, showing the different horizons to the top of the sub-horizon B_4 (105 cm deep). (C) Final result of the Antampolo pit Q2 (S14º43.468' - E050º12.860'), 140 cm deep. (D) Sample from the A horizon of the Andranotsara pit Q1, it includes fungi-bearing resin beads, humus-quartz aggregates, some roots, quartz grains and pellets. (E) Sample from the sub-horizon B_1 of the same pit Q1, formed mainly by non-rounded quartz grains, and a small number of fungi-bearing resin beads. (F) Resin remains formed under aerial conditions, found in the Antampolo pit Q2 in the A horizon, showing the intense pulverulent surface alteration. (G) Sample from the sub-horizon B_3 of the Antampolo pit Q2, formed by non-rounded quartz grains with clay. Notations: r = *H. verrucosa* root; O, A, B_1 to B_4 = horizons and sub-horizons.

The soil of Andranotsara pit Q1 (Figs 4A–B) developed in an ancient coastal sand dune resulting in a low level of moisture and nutrients. The pit Q1 was dug delimited by three ancient *Hymenaea* trees stumps (82, 91 and 94 cm of trunk diameters). The trees were affected by two hurricanes, one in 2000 and one in March 2017, and they were cut down leaving their root systems. The soil was mainly sandy, uncemented and with a high degree of drainage.

Excavation-depth reached 175 cm (Fig 5A). O horizon: 2–3 cm with humus on ground surface, grass roots and fungi-bearing resin beads (named above as “soil beads”; see the results in this section below), and fragments of resin pieces formed under aerial conditions (on branches or on the trunk). A horizon: the top of the soil was a very dark level, rich in organic matter, with a thickness of 10–15 cm that presents many roots of grass, little fragments of *Hymenaea* leaves, abundant fungi-bearing resin beads (Ø 1.9–5.5 mm) (Fig 4D), and some fragments of resin (formed under aerial conditions, < 3 mm). The inorganic fraction is composed mainly by sand rich in quartz. B horizon: the subsoil was divided in four sub-horizons. The sub-horizon B_1, 25–30 cm thickness, was composed of transparent quartz sand with some organic matter. Its upper part also contains some fungi-bearing resin beads (Ø <1.1 mm, Fig 4E) and isolated pieces of aerial resin observed already in the lower part of A horizon. Sub-horizon B_1 is a level with many *Hymenaea* roots, some with resin wraps that agglutinate the sand. Sub-horizon B_2 is a 60–70 cm interval of ocher sand, very monotonous, with unrounded grains, having some roots but with very little organic matter and resin (Fig 4B). Resin and sand aggregates are present around the roots at 60 cm depth (Figs 7A–B). At this level, the clay content is high, however, it does not form aggregates. A few grains of igneous rocks of greenish white color are also present. At 50 cm deep, the last pieces (very few) of resin preserved are located; the ocher tone of B_2 increases towards sub-horizon B_3, which is a 2 cm dark ocher sandy level with some white-greenish grains of igneous rocks; from 2 to 5 cm there is a change in the color of the sand to lighter ocher and later to gray with dark spots. Sub-horizon B_4, with a minimum thickness of 57 cm, is composed by very clear monotonous sand and apparently lacks roots.

Fig 5. Pedologic logs of the pits excavated in the Sambava region: (A) Andranotsara pit Q1, 170 cm deep, and (B) Antampolo pit Q2, 140 cm deep. The detailed explanation is in the text. Pits geographical location in Fig 4. (C) FTIR analyses of three samples in order to determine the origin of soil beads (= fungi-bearing resin beads) preserved in the O horizon of the forest soil. The analyses include samples of resin of 1. soil beads from the soil surface in Antampolo,

another place; 2. soil beads of the O horizon of the Antampolo pit Q2, and 3. living *H. verrucosa* tree resin in Antampolo.

The Antampolo pit Q2 (Fig 4C) on the side of a hill developed above a ferralitic soil on top of a plutonic rock (observed laterally, not in the area where the pit developed). The pit Q2 was dug around two *Hymenaea*'s tree stamps (80 cm and 100 cm of trunk diameters) unrooted and cut in 2000 after a hurricane. Excavation-depth reached 135 cm (Fig 5B). O horizon: 3 cm of quartz-sand with high proportion of humus and grass roots, abundant fungi-bearing resin beads and aerial pieces of degraded resin. Neither pod resin beads nor isolated seeds of *H. verrucosa* are observed, perhaps due to rapid decomposition. A horizon: very dark level, rich in organic matter, with a thickness of 6 cm that presents abundant roots millimetric in section, fungi-bearing resin beads (Ø 1.2–5.4 mm), and some big pieces of resin (some of them developed under aerial conditions, > 5.2 mm) (Fig 4F). B horizon: the subsoil is divided in four sub-horizons. Sub-horizon B_1, with a thickness of 24–26 cm, is composed by dark gray fine sand with some organic matter and little roots. Its upper part also contains some fungi-bearing resin beads (Ø < 1.5 mm) and big fragments of highly altered resin (some related with aerial exudations), not present in the lower part. In the interval of 20 to 30 cm depth, resin crusts occur associated with roots (some of them highly degraded, others with vitreous interior, orange in color). Sub-horizon B_2 is composed by ocher sand very monotonous with clays non-forming agglutinates (Fig 4G) with a thickness of 60 cm. There is no presence of resin in any of its forms. Sub-horizon B_3 is composed exclusively by fine sand quartz without clay, with a minimum thickness of 40 cm. C horizon was not reached.

In the north, in the Antsiranana region, after an intense search, no copal or resin deposit in the margins of the rivers (point bars, oxbows), beaches, or deltas related to the drainage of the *Montagne d'Ambre* were found (Figs 6A–D). We consider that this region lacks copal outcrops.

~~More probably~~ publications mentioning *Montagne d'Ambre* as the source of "Madagascar copal" (i.e., Lourenço, 2000; Poinar et al., 2001, or websites such as Tower Gallery, 2019) followed uncritically the unconfirmed information provided by native traders, possibly due to spurious interests, because in that region the "Madagascar copal" is neither produced nor marketed. *Hymenaea verrucosa* does not grow in the *Montagne d'Ambre* region because of its evergreen tropical forest microclimate. In the drier coastal region of Antsiranana - Sakaramy *H. verrucosa* is only represented by scattered individuals, not forming forests, nowadays. The edaphic profiles on which *H. verrucosa* lives are ferralitic on basalts (rhodic ferrasols sensu Jones et al., 2013). Here pits in soil were not dug, but profiles were observed on the margins of the rivers and in some parts of the delta plains of the rivers that limit Antsiranana (Figs 6C–D) that drain the *Montagne d'Ambre* area (going through the Sakaramy area) to the sea. O horizon: 2–3 cm with humus on ground surface with grass roots without evidence of resin particles. A horizon: a brown clay-sandy level poor in humus, but with numerous roots and with a thickness around 10 cm; neither fungi-bearing resin beads nor pieces of resin developed under aerial conditions have been observed. The first one meter of the B horizon is composed by low-consolidated clays, coarse-textured, brown to red wine color; the next one and a half meters is a coarse-textured brown–red claystone more consolidated than above; in the first few centimeters of this horizon the amount of organic matter does not exceed 2%. C horizon: light brown, weathered rock starting abruptly with some reddish spots in places. Spheroidal weathering (small balls) because of the chemical alteration along intersecting joints is observed. Both resin and copal are not observed in any ferralitic soil studied. At the western Antsiranana we observed some chromic luvisols, and lithic leptosols in the eastern part, both also lacking resin accumulations. In this area we collected resin directly from the trees; *Hymenaea* is not abundant in the Antsiranana region, it has a mystic significant for the local people, and, at least today, trees are dispersed in the region but not forming forests. Commerce of resin or copal is unknown in this region.

Fig 6. Soils where *H. verrucosa* grows. (A) Ferrosols in Sacaramy (between *Montage d'Ambre* and Antsiranana). (B) Searching for resin in the litter around *H. verrucosa* trunk, rooted in a ferrosol, in Sacaramy. (C) Ferrosols in the delta of the river Antomboka, in Anamakia next to the salt industry of Antsiranana. (D) Ferrosols in a delta plain in front of Nosy Lonjo (Andovobazaha Bay, Antsiranana). (E) Regosols/arenosols in the Pangalanes Chanel margins, Ambahy (Nosy Varika, Mananjary). (F) Arenosols in a lake margin between Nosy Varika and Ambahy.

In Mananhary (Nosy Varika - Ambahy) area, the edaphic profiles are similar as in Sambava -Antalaha (Andranotsara - Antampolo). Here no pits were dug, but soils were observed in the margins of the Pangalanes Channel (Figs 6E–F) and lakes. No resin accumulation levels were observed, only a few pieces of resins in O and A horizons of dystric regosols/arenosols. Commerce of *Hymenaea verrucos*a resin is common in this region.

Hymenaea lives rooted in sandy soils in areas with a high rainfall rate, where the soils are intensely drained. This drainage means that, in a relatively short time, the organic matter of the soil including resin inputs disappears, mainly by decomposition and degradation (Daher and Bellot-Gurlet, 2013b). Our investigations in soils of different areas in Madagascar with presence of *Hymenaea* trees indicate that the high rate of rain combined with high degree of drainage strongly promote the decomposition of the resin at deeper layers than 50 cm. Resin cannot be preserved in highly washed lateritic soils because they are characterized by their scarcity in silica and their high amount of iron, alumina and/or other non-removable minerals, constituting a highly oxidizing medium incompatible with preservation of labile organic matter as resin. Burton (1860) and Sunseri (2007) mentioned that "Zanzibar copal" in Tanzania was dug in red lateritic soils, where *Hymenaea verrucosa* had lived several thousand years ago. However, we did not find copal or resin in Malagasy ferralitic soils. Burton (1860) mentioned that according to Arab traders in Tanzania, the redder the soil, the better was the copal for the market. He described that the level at which the "copal" was extracted in Tanzania corresponded to a dark level of humus

and hardened clay that varied from a few centimeters to 45 centimeters in thickness, covered by a thin layer of white sand.

In the Malagasy regions we explored, no level of white sand covered the subsurface level, which is rich in organic matter; no level rich in organic matter has been found in the subsoil under several decimeters of sand. Similar levels rich in organic matter, from which the Tanzanians extracted "copal", are present in Madagascar in the first 15 cm from the surface, forming the most superficial horizons of the Malagasy forests soils. In this level most of the resin samples have been found. This suggests that resin pieces in other East African regions were possibly protected and preserved by clay sand levels at several decimeters' depth, promoting the formation of copal deposits. However, this seems to be not the case in Madagascar.

According to Lewton (1899), the "fossil copal" ("Zanzibar copal" from Tanzania) sometimes showed a crust of oxidized material (known as "goose skin") when it was removed from the ground. This oxidized material consisted of large to small excrescences due to weathering (Dieterich, 1920), nevertheless, the interior remained transparent and clear, and varied from pale yellow to brownish in color. During the field investigations we did not find this kind of crust in resin pieces from the pits; pieces found in the subsoil externally showed a pulverulent, fine white coat (Fig 7G) most likely produced by the action of resinicolous microorganisms (Tibell and Titov, 1995; Speranza et al., 2015) and/or weathering.

Fig 7. *Hymenaea verrucosa* fungi-bearing resin beads found abundantly in some pit horizons (figured specimens from pit Q2, Antampolo) as important resin inputs. (A–B) Fungi-bearing resin beads as organic matter isolated from the sand. (C) Fungi-bearing resin bead sectioned showing the small spherical, organic structures purple in color interpreted as fungal sclerotia. (D) SEM image of the same sectioned bead. (E) SEM detail of the inner cavity containing sclerotia and inset showing a detail of the their punctated external surface. (F) SEM detail of the external unaltered layer (left) and the state of preservation in the interior of the resin body of the fungi-bearing bead (right lower part), see inset indicated in D. (G) SEM detail of the resin body of the bead invaded by a fungal mycelium (see inset indicated in D), which is of the same fungus that produced the sclerotia, most likely a representative of the genus *Aspergillus*.

In the O and A horizons of both pits, abundant millimetric rounded pieces invaded by fungi have been obtained (Figs 4D, 7A–B). These pieces are very abundant in the soil of the studied areas, and for example are abundantly present in the surface of the crops in these areas. FTIR analyses agree with *H. verrucosa* resin (Fig 5C) (fungi-bearing resin beads); the fungi-bearing resin beads are not resin contained in the wall of the fruit pods (Fig 2E), which remained on the soil surface as the fruit pods decomposed, as initially hypothesized. Optical microscopy and SEM showed that the core is a resin-free gap (like a big bubble) containing small spherical (Ø ca. 150 µm), organic structures reddish brown in color (Figs 7C–E), which does not match the resin beads of the pods (Fig 2E), since the latter are of solid resin (herein named pod resin beads). The fungi-bearing resin beads have a homogeneous shape and are invaded by Recent fungal mycelia except for a fine external layer (Figs 7F–G), with the internal "bubble" containing the small spherical, organic structures that have been interpreted as sclerotia of the same invasive fungus (Figs 7C–E). The taxonomic identification of yeast and fungi present in~~to~~ the fungi-bearing resin beads by means of 16S rRNA gene sequencing (Next-Generation Sequencing), using a combination of culture-dependent and independent techniques, is in progress and will be published elsewhere. Preliminary results indicate that most likely the filamentous fungus belongs to the genus *Aspergillus*, and that taxonomic determination fits well with the presence of the sclerotia in the beads and their morphology (Abu El-Souod et al., 2017). The structure of the mycelium present in the resin body is similar to some resinicolous fungi in gimnospermous resin, both extant (Beimforde and Schmidt, 2011, and own observations in New Zealand samples) and fossil (Speranza et al., 2015). These fungi-bearing resin beads seem to be small resin emissions from the fine, small roots of *Hymenaea*. Their presence in soil O and A horizons can be probably explained by the observed abundance of roots of different sizes in the more superficial horizons. We consider that the preservation of these resin inputs is because the tree produces them up to its death at a certain soil depth from its roots, thus the fungi-bearing

resin beads are the most abundant and recent resin inputs present in the forest soil ~~and, for that~~ reason, they are not completely degraded in the studied soils, ~~this~~ despite the fact that the presence of dense mycelia invading the resin bodies most likely promotes the degradation of these resin pieces, not the other way round. ~~If~~ the fungus induced ~~or not~~ the resin production from the roots by infection and became embedded (obtaining an extra protection of their sclerotia), maybe providing a benefit to the tree~~, or if the~~ fungus ~~is~~ resinicolous and simply uses the resin as a food source (many species of *Aspergillus* demonstrate oligotrophy)~~, between other potential scenarios, are currently open questions~~. In any case, from a taphonomical point of view, these peculiar beads have been observed as important inputs of resin in the *Hymenaea* forest soils of Madagascar.

Age of the "Madagascar copal"

The "Madagascar copal" is sold around the world, mainly because it contains arthropods preserved (Figs 8C–D); due to its brightness, usually merchants say that it is polished copal, but actually it consists of hardened resin directly harvested from trees (Fig 8D). Both amber and copal originally were resin pieces that became buried and experienced fossil diagenetic processes (Martínez-Delclòs et al., 2004); they differentiate from their respective original resin due to their highest polymerization grade (see section 2.1). As we explained above, we follow the proposal of Anderson (1996), based on objective analysis, who presented a scale based on ^{14}C dating.

Fig 8. Pieces of resin obtained from a tree and from the pits at several depths. (A) Resin exuded by roots that form quartz-grains aggregates around (crusts) as found in the B horizon, Andranotsara pit Q1. (B) Thin section of the A sample; the label "root" shows the position of the *H. verrucosa* root; the resin (brown) when exuded by the root agglutinated the quartz sand grains (transparent). (C) Resin flow of *H. verrucosa* with arthropods embedded, Ambahy (Mananjary). (D) Detail of the same flow showing the original transparency, as if polished. (E) Sample of the

resin found in Andranotsara pit Q1 at sub-horizon B_1 between 15–30 cm depth. (F) Sample of the resin found in Antampolo pit Q2 at sub-horizon B_1 between 20 to 30 cm depth. (G) Sample of the resin found in the same pit at sub-horizon B_2 at 50 cm depth. E–G are samples ^{14}C analyzed.

Burleigh and Whalley (1983), based on studies of radiocarbon-dating of "copal" pieces that came from Tanzania and Kenya (without precise localities), suggested that their ages are not older than 100 years. Poinar (1999) and Poinar et al. (2001) mentioned that "Madagascar copal" (also without precise locality or localities) can be between 50 and 100 years old.

Results are reported in pMC (percent modern carbon) when the analyzed material had more ^{14}C than did the modern (AD 1950) reference standard, indicating that those samples of "Madagascar copal" were exuded in the last 60 years (pMC) in calibrated calendar years (cal BC/AD) or in Conventional Radiocarbon Ag (BP) when the material had less ^{14}C than did the modern reference standard. The samples radiocarbon dated are the following:

Sample	pMC	BP	Cal. AD/ BP	Comments
Madagascar copal	105.20 ± 0.3			Two pieces of "Madagascar copal" radiocarbon dated in June 2012 by Dr Thomas Hörnschemeyer.
Andranotsara close to pit Q1 collected at 50 cm		300 ± 30		Resin collected in a hole made by native people close to the Andranotsara pit Q1, in which resin was not found deeper than 50 cm.
"Zanzibar copal" SMF Be 3724		80 ± 30	Cal AD 1710 to 1720 / Cal BP 240 to 230	The analysed piece arrived at the Senckenberg Research Institute in Frankfurt in 1901.
Andranotsara pit Q1 at 15–30 cm depth	140.47 ± 0.52			Fig 8E
Antampolo pit Q2 at 20–30 cm depth		180 ± 30		Fig 8F
Antampolo pit Q2 at 30–70 cm depth	180.83 ± 0.41			Fig 8E

Table 1. Characteristics and results of the samples ^{14}C analyzed. Last four samples were analyzed by Beta Analytics.

Our analyses of ^{14}C, conducted on these diverse samples from museum pieces and pieces obtained from the pits, suggest that the "Madagascar copal" has little age variation.

The decomposition of the litter can occur rapidly in tropical forests, mainly due to the activity of a great diversity of microbes, fungi and invertebrates, this together with high humidity and temperature (Razanamalala et al., 2017). In the *Hymenaea* forest the organic matter, including resin, in a sandy soil, with high a percentage of annual rainfall, the presence of termites, ants, and other decomposers, remains preserved only for short time on the surface (Powers et al., 2009; Krishna and Mohan, 2017) being almost absent below a few decimeters depth. We observed in the pits that fungi-bearing resin beads (Ø <6 mm) disappeared completely below a few centimeters' depth. Apparently, all organic matter is intensely recycled in a few years, including resin, due to the soil washing processes during the seasonally rainy periods and due to the action of decomposing microorganisms, possibly including resinicolous fungi. During dugs in 2017 in Andranotsara, pit Q1, and in Antampolo, pit Q2, aerial resin pieces under 20 cm depth were very scarce.

Radiocarbon-dating indicates a maximum period of 300 years for the complete degradation of the *Hymenaea* resin fragments in the Malagasy subsoil. One piece of resin obtained by us at 50 cm depth in the subsoil has arthropod bioinclusions, however, their degree of preservation (Figs 8E–G) is notably poorer than in the case of pieces collected directly from the trees (Figs 8C–D) and then sold in markets. All pieces collected from the subsoil have a white coat of alteration, as already mentioned, most likely produced in part by fungi and bacteria or weathering giving a matte, pulverulent aspect (Fig 8F). This probably is responsible for resin destruction over time. In the samples less altered, the inner part or core remains vitreous and orange in color.

The pieces of "Madagascar copal" rich in biota preserved as bioinclusions and marketed came from the Sambava or Mananjary regions, where resin is harvested and not obtained from the subsoil. Interviews at the different regions revealed that Sambava, Antalaha and Mananjary are the commercial centers of this material, which is collected by people from the different towns in the surrounding areas and sold to main traders in an informal market. Resin is collected over the year as an extra activity beside other commercial activities such as the vanilla harvest, produce charcoal, etc. The best commercial samples are those collected directly from the trees and not from the soil because of its ~~bright~~ aspect and insect content. This dynamic, domestic trade provides the most important part of the resin sold as “Madagascar copal”, if not all.

All these observations and results demostrate a recent age for the "Madagascar copal", commonly having bioinclusions, thus a commercial misinterpretation occurs with respect to one of its more important putative features; therefore, they should be considered as a modern resin and not as an ancient resin.

Paleobiological implications for previous “Madagascar copal” studies

The first scientific work on arthropods included in "East African copal", but registered as “Zanzibar copal”, studied a spider (Bloch, 1776; Dunlop and Jekel, 2008). Until the 16th century most of the resins or copals regardless of their origin in East Africa (possibly but not certainly including Madagascar), were denominated in the markets and in the scientific works as "Zanzibar copal" (or gum copal), "Calcutta copal", "Bombay copal", or "India copal" (Meunier, 1909). Nevertheless, all these “copals” corresponded to different qualities of the "Zanzibar

copal", which was sold in Indian markets (Filhol,1842–43) with origin in East Africa and produced by *H. verrucosa*.

It is not until the end of the 19th century that the first scientific works cited Madagascar as the country of origin of this type of "copal" (De Saussure, 1890; Fairmaire, 1894). De Saussure (1890) described an endemic bee from “Madagascar copal”, *Liotrigona madecassa* (Saussure, 1890) that lives today in the western part of the island (Koch, 2010; Engel and Michener, 2013). At the end of the 20th century, due to the commerce worldwide of the “Madagascar copal”, a plethora of arthropods attracted the attention of entomologists and paleoentomologists mainly for their country of provenance – a hotspot of biodiversity – initiating a serial of studies lacking crucial data with respect to the geological context(s), the location of the outcrop(s), and the age of the pieces. Some of these species described from “copal” corresponded to living species that were previously described (i.e., Bosselaers et al., 2010; Vankerkhoven et al., 2010), mainly from the coastal forests of Africa. However, other species are only known preserved in "copal" (i.e., Meunier, 1909; Bosselaers, 2004; Lourenço, 2009), thus it is possible that some of them could be found alive in the Malagasy eastern coastal forests (Bosselaers et al., 2010; Guilbert and Heiss, 2016). Recently, Riquelme et al. (2015), after the study of scorpions from the Miocene Mexican amber, proposed a phylogeny that included three buthid species found in the “Madagascar copal”; one species was assigned to the Recent genus *Microcharmus* (Microcharmidae) and the rest undetermined species to the Recent genus *Palaeogrosphus* (Lourenço, 1996, 2009; Lourenço and Henderickx, 2012). They suggested that “Malagasy copal” is less than 250 years old (without explanation or new data), and that the species recognized, not fossil or sub-fossil species, may be considered as extant species. The absence of dating of the pieces of “Madagascar copal”, including their bioinclusions, is a recurrent and never discussed topic in taxonomic publications. This circumstance triggers problems in other research fields such as

biogeography (i.e., Fisher, 1997; Griswold et al., 2012; Beron, 2018), phylogeny (i.e., Harms and Dunlop, 2017; Heikkilä et al., 2018) or molecular clocks (Büsse et al., 2017).

It can be assumed that species widespread in Madagascar can be recognized in "copal", however, some taxa can have become extinct because of habitat alteration and/or destruction. Some authors assumed that the "Madagascar copal" has a wide temporal range, suggesting that the oldest samples may include extinct species, while youngest samples may include species that are still alive (Bukejs and Legalov, 2017). Labandeira (2014) considered that the East African copal" (with Tanzanian and Malagasy origin) is a sub-fossil resin 0.7–0.05 Ma old, produced in a subtropical mangrove forest including a combination of recently extinct and extant congeneric species, showing an historical structure of modern tropical ecosystems. However, *H. verrucosa* is not part of the mangrove forest in Madagascar or in Tanzania, an ecosystem highly developed on both margins of the Mozambique Channel.

All paleobiological works must include the age of the deposit where the studied taxa were found. In the case of the "Madagascar copal" all the newly described species have been inaccurately located. All studied specimens (between 100 and 120 different species, in about 80 publications) were described under the generic name "Madagascar copal" and several provenance areas have been proposed. That imprecision is also especially problematic for paleobiological/biological studies, taking into account that *H. verrucosa* lives along 2,000 km of the east coast of Madagascar and in diverse ecosystems (see section 3.1). Thus, studied specimens can originate up to thousands of kilometers away from each other. It is also highly probable that mixed specimens from different localities are present in both retail shop and in collections, so putative paleobiological and/or paleoenvironmental studies could be based on unsuitable data.

For most of the pieces obtained before the beginning of the 20th century it is virtually impossible to know if their provenance is the East African countries, such as Tanzania,

Mozambique, or Madagascar, because all were sold under "Zanzibar copal" or "East African copal" names. This absence of accurate geographical information implies that the studies or inferences on biogeography, ecology, and/or loss/change of biodiversity, among other topics, are inaccurate. This is the situation until a technique to recognize the geographical origin of unlabeled or inadequately labeled resin pieces is available.

Conclusions

Hymenaea trees occur in the north and in the east margins of Madagascar Island. They are most abundant in the regions of Vohemar, Sambava, Masoala, and in the southeast at Mananjary (see also Missouri Botanical Garden, 2019). Today, resin with arthropod bioinclusions is collected from living resin-producing *Hymenaea verrucosa* trees, by people that sell it in small quantities.

From our studies "Madagascar copal" cannot be consider as sub-fossil resin, but as Recent resin a, circumstance that clearly-explains the occasional presence of ~~some~~ living arthropod species embedded in it. "Madagascar copal" containing remains of preserved organisms housed in museums and in private collections corresponds to Recent resin and therefore does not have the paleontological relevance that has been attributed to it, however, it is still relevant for actuotaphonomic research. This circumstance requires the revision of the described taxa to avoid both taxonomic errors and inaccurate paleoenvironmental reconstructions. Most likely, the species that have been described exclusively from the "Madagascar copal" have not been searched for in the proper habitat or cannot successfully be trapped by conventional entomological traps (Solórzano Kraemer et al., 2015, 2018). They probably can be found in the coastal lowland forests, where *H. verrucosa* grows, and not from the evergreen tropical forest of the island. However, this observation is based on the premise that the forests have not yet been

strongly distorted or even deforested, because Madagascar is one of the most endangered habitats in the world.

There is a gap of information about how "copal" or resin was extracted in Madagascar during the 18th and 19th centuries; only indirect information comes from ship captains or military who reported local trade observed during their trips, or from merchants looking for material for the varnish industry. ~~The~~ "Madagascar copal" was known for centuries in the harbors of the Indian Ocean under the trade name "copal of Zanzibar", later as "Madagascar copal"; however, if it was excavated historically, the extraction localities remain unknown. More probably it was exploited by the same system as nowadays. Thus, it was collected directly from trees or at shallow depths in the ground, and/or when holes were excavated to produce charcoal.

The most ancient resin pieces found in soils by us are only 300 years old; they were found at < 50 cm depth and considerably altered (resin has not been found deeper). In consequence, the genesis of the Miocene amber deposits in Mexico and Dominican Republic in which the *Hymenaea* ssp. resin fossilized, had to be different from those that now exist in the Recent African habitats where the most primitive species of *Hymenaea* lives, and from which all the neotropical species derived. Investigations of the formation of amber deposits in Central America, and copal deposits from Colombia and Dominican Republic, could be of great relevance.

It is not ruled out that amber-bearing and/or sub-fossil resin-bearing deposits exist in Madagascar that originated from ancient exudations of *H. verrucosa*. However, the results obtained from the geologically/edaphically studied deposits/soils, the absence of geological deposits exploited commercially, and the modern regular collection of *H. verrucosa* resin containing arthropods and other biota for sale, suggest their absence. We propose that, after this study, any new taxon described from putative "Madagascar copal" must be accompanied by a ^{14}C analysis of the material in which the bioinclusion is present, at least respect to the holotype

to establish its validity. For obvious reasons, museums with Malagasy pieces of supposed copal containing type specimens should undertake a review of them by performing ^{14}C analyses.

Importantly, therefore, even if "Madagascar copal" is a Recent resin produced during the Anthropocene, because it could contain extinct species due to the high rate of deforestation in the last 300 years. Intense current deforestation continues, and perhaps in the next decades the biota included in Recent *H. verrucosa* resin, together with the historical entomological collections, will be the only reservoirs of knowledge to investigate a part of the entomofauna of the fragile lowland tropical forests of Madagascar.

Acknowledgements

The authors are grateful to R. Ravelomanana, M. Asensi, S. Rahanitriniaina, T. Rakotondranaivo, M. Madiomanana, and J. Andrianabo for their assistance and help during the scientific fieldworks, and J.L. Totovanona and G. Jahovelo for authorization to dig in the forests of their property. We thank Dr T. Rakotondrazafy, Director of the Départ. de Paléontologie et Anthropologie Biologique, and Dr E.M. Randrianarisoa, Director of the Départ. d'Entomologie, both in the Université d'Antananarivo, for their help and advice with administrative arrangements. We thank also Dr N. Ferrer from the UB Scientific Facilities for the FTIR analysis help, R. Kunz (Senckenberg Research Institute and Natural History Museum Frankfurt) for the digitalization of figures 1 and 5, Dr T. Hörnschemeyer for providing the C^{14} analysis of one of the analyzed pieces, and Alan Lord, both from the same institution for English help. Furthermore, the authors wish to acknowledge the team of the Malagasy Institute for the Conservation of Tropical Environments (ICTE/MICET) for their assistance in aspects of the administrative development of our work in Madagascar. The Ministère de l'Environnement, de l'Écologie et des Forêts give us the permissions to work in the Malagasy protected areas

(192/13/MEF/SG/DGF/DCB.SAP/SCB; 060/15/MEEF/SG/DGF/DCB.SAP/SCB, and 192/17/MEEF/SG/DGF/DSAP/SCB.Re). We thank the Spanish Ministry of Economy, Industry and Competitiveness (Project CGL2014-52163), the Spanish Government (Project AEI/FEDER, UE CGL2017-84419), the National Geographic Global Exploration Fund Northern Europe (Project GEFNE 127-14), and the German VolkswagenStiftung (Project N. 90946), for financial support.

Competing interests’ statement

The authors declare that there is no conflict of interest regarding the publication of this article.

Author contributions

Conceptualization: XD, EP, MSK; Methodology: XD, EP, MSK; Validation: All; Formal Analysis: XD, EP, MSK; Investigation: XD, EP, MSK; Resources: All; Writing-Original draft: XD; Writing-Review & Editing: All; Visualization: XD, EP, MSK; Funding Acquisition: XD, MSK.

References

Abu El-Souod, S., Awadalla, O.A., Assawah, S.M.W., Mahmoud, Y.A.G., El-Debaiky, S.A.E.K. Studies on the sclerotia of some species in the genus *Aspergillus*. Egyptian Journal of Botany. 2017; 57:395–404.

Ali, J.R., Huber, M. Mammalian biodiversity on Madagascar controlled by ocean currents. Nature. 2010; 463:653–657.

Anderson, K.B., Crelling, J.C. Introduction. In: Anderson, K.B., Crelling, J.C. (Eds.), Amber, Resinite, and Fossil Resins, American Chemical Society Symposium Series, No. 617, American Chemical Society. 1995; Washington, DC, pp.1–7.

Anderson, K.B. The nature and fate of natural resins in the geosphere – VII. A radiocarbon (^{14}C) age scale for description of immature natural resins: an invitation to scientific debate. Organic Geochemistry. 1996; 25:251–253.

Beaujard, Ph. East Africa, the Comoros Islands and Madagascar before the sixteenth century: on a neglected part of the world system. Azania. 2007; 42:15–35.

Beimforde, C., Schmidt, A.R. Microbes in resinous habitats: A compilation from modern and fossil resins. Advances in Stromatolite Geobiology Lecture Notes in Earth Sciences. 2011; 131:391–407.

Beron, P. Factors Determining the Distribution of Arachnida. Zoogeography of Arachnida. Monographiae Biologicae. 2018; 94:59–134.

Blanchard, R.A.E., Boule, M., Delhorbe, C., del Castillo, E.D., Froidevaux, H., Grandidier, G. et al. Madagascar au début du XX siècle. Société d'éditions scientifiques et littéraires, 1902; 465 pp., Paris.

Bloch, M.E. Beytrag zur Naturgeschichte des Kopals. Beschäftigungen der Berlinischen Gesellschaft Natur- forschender Freunde. 1776; 2:91–196.

Bosselaers, J. A new Garcorops species from Madagascar copal (Araneae: Selenopidae). Zootaxa. 2004; 445:1–7.

Bosselaers, J., Dierick, M., Cnudde, V., Massachaele, B., Van Hoorebeke, L., Jacobs, P. High-resolution X-ray computed tomography of an extant new *Donuea* (Araneae: Liocranidae) species in Madagascan copal. Zootaxa. 2010; 2427:25–35.

Bruneau, A., Klitgaard, B.B., Prenner, G., Fougere-Danezan, M., Tucker, S.C. Floral evolution in the Detarieae (Leguminosae): Phylogenetic evidence for labile floral development in an Early-Diverging Legume Linage. International Journal Plant Science. 2014; 175:392–417.

Bukejs, A., Legalov, A.A. New Species of Sub-Fossil Weevils (Coleoptera, Curculionidae) in Madagascar Copal. Paleontological Journal. 2017; 51:196–202.

Burgess, N.D., Clarke, G. Ph., Rodgers W.A. Coastal forests of eastern Africa: status, endemism patterns and their potential causes. Biological Journal of Linnaean Society. 1998; 64:337–367.

Burgoyne, P.M., van Wyk, A.E., Anderson, J.M., Schrire, B.D. Phanerozoic evolution of plants on the African plate. Journal of African Earth Sciences. 2005; 43:13–52.

Burleigh, R., Whalley, P. On the relative geological ages of amber and copal. Journal of natural History. 1983; 17:919–921.

Burton, R.F. The Lake Regions of Central Africa. A picture of exploration. Harper and Brothers Publishers. 1860; 572 pp., New York.

Büsse, S., von Grumbkow, Ph., Mazanec, J., Tröster, G., Hummel, S., Hörnschemeyer, Th. Note on using nuclear 28S rDNA for sequencing ancient and strongly degraded insect DNA. Entomological Science. 2017; 20:137–141.

Campbell, G. Indians and Commerce in Madagascar 1869-1896. African Studies Seminar Paper, University of the Witwatersrand. African Studies Institute. 1993; 345:1–21.

Chami, F.A. Early ironworking communities on the East African coast: excavations at Kivinja, Tanzania. In: Kusimba, S., Kusimba, C. (Eds.), East African Archaeology: Foragers, Potters, Smiths, and Traders, University of Pennsylvania Press. 2003; Philadelphia, pp. 87–97.

Chidumayo, E.N., Gumbo D.J. The Dry Forests and Woodlands of Africa. Managing for Products and Services. Earthscan, Center for International Forestry Research. 2010; 288 pp., London & Washington D.C.

Clarke, G.P. Defining the eastern African coastal forests. In: Burguess, N.D., Clarke, G.Ph. (Eds.), Coastal Forests of Eastern Africa. 2000; Gand, pp. 9–26.

Clifford D.J., Hatcher P.G. Structural transformations of polylabdanoid resinites during maturation. Organic Geochemistry. 1995; 23:407–418.

Creamer, P.M. A Comparison of Resinous Artifacts in the Ancient Near East. The Ohio State University. Department of Anthropology Honors Theses. 2014, 74 pp. accessible in the URL: http://hdl.handle.net/1811/60326

Crottini, A., Madsen, O., Poux, C., Strauss,A., Vieites, D.R., Vences, M. Vertebrate time-tree elucidates the bio-geographic pattern of a major biotic change around the K-T boundary in Madagascar. Proceedings of the National Academy of Sciences USA. 2012; 109:5358–5363.

Crowther, A., Veall, M.-A., Boivin, N., Horton, M., Kotarba-Morley, A., Fuller, D.Q. et al. Use of Zanzibar copal (*Hymenaea verrucosa* Gaertn.) as incense at Unguja Ukuu, Tanzania in the 7-8th century CE: chemical insights into trade and Indian Ocean interactions. Journal of Archaeological Science. 2015; 53:374–390.

Cunnigham, A., West, P.R., Hammond, G.S., Langenheim, J.H. The existence and photochemical initiation of free radicals in *Hymenaea* trunk resins. Phytochemistry. 1977; 16:1442–1443.

Daher, C., Bellot-Gurlet, L. Non-destructive characterization of archaeological resins: seeking alteration criteria through vibrational signatures. Analytical Methods, Royal Society of Chemistry. 2013; 5:65–83.

Daher, C., Bellot-Gurlet, L., Le Hô, A.S., Paris, C., Regert, M. Advanced discriminating criteria for natural organic substances of Cultural Heritage interest: Spectral decomposition and multivariate analyses of FT-Raman and FT-IR signatures. Talanta. 2013; 115:540–547.

De Cordemoy, H.J. Les Plantes à Gommes et à Resines. O. Doin et Fils, Editeurs. Encyclopédie Scientifique. 1911; 412 pp., Paris.

De Gouvenain, R.C., Silander, J.A. Do Tropical Storm Regimes Influence the Structure of Tropical Lowland Rain Forests? BioTropica. 2003a; 35:166–180.

De Gouvenain, R.C., Silander, J.A. Littoral Forest. In: Goodman, S.M., Benstead, J.P. (Eds.), The Natural History of Madagascar, University of Chicago Press, Chicago. 2003b; pp. 103–109.

De Saussure, H. Histoire naturelle des Hyménoptères. In: Grandidier, A. (Ed.), Histoire Physique, Naturelle et Politique de Madagascar 20, Imprimerie Nationale. 1890 [1892]; xxi + 590 pp., +27 pls., Paris [NB: date of publication is likely 1892].

Dieterich, K. The Analysis of Resins, Balsams and Gum Resins with a Bibliography. Scott, Greenwood & Son. 1920; 431 pp., London.

Dikow, T., Bayless, K.M. Taxonomic revision of the genus *Schildia* Aldrich, 1923 (Diptera: Asilidae: Leptogastrinae) with the description of new extant and extinct species. Ins Sys Evo. 2009; 40:253–289.

Drzewicz, P., Natkaniec-Nowak, L., Czapla, D. Analytical approaches for studies of fossil resins. Trends in Analytical Chemistry. 2016; 85:75–84.

Dunlop, J., Jekel, D. The oldest available fossil arachnid name. Palaeodiversity. 2008; 1:87–92.

Elton, F. 1874. On the Coast Country of East Africa, South of Zanzibar. J Roy Geo Soc. Lond. 1874; 44:227–252.

Engel, M.S., Michener, Ch.D. A minute stingless bee in Eocene Fushan amber from northeastern China (Hymenoptera: Apidae. J Mel. 2013; 14:1–10.

Fairmaire, L.M.H. Coléoptères de l'Afrique intertropicale et australe. Annales de la Société entomologique de Belgique. 1894; 38:314-335.

Federman, S., Dornburg, A., Downie, A., Richard, A.F., Daly, D.C., Donoghue, M.J. The biogeographic origin of a radiation of trees in Madagascar: implications for the assembly of a tropical forest biome. BMC Evolutionary Biology. 2015; 15: 216 (11 pp.).

Feist, M., Lamprecht, I., Müller, F.M. Thermal investigations of amber and copal. Thermochimica Acta. 2007; 458:162–170.

Filhol, M. On Indian Copal. Phar J Trans. 1842–43; 2:773–774.

Fisher, B.L. 1997. Biogeography and ecology of the ant fauna of Madagascar (Hymenoptera: Formicidae). Journal of Natural History. 1997; 31: 269–302.

Fougère-Danezan, M. Phylogénie moléculaire et morphologique des Detarieae résinifères (Leguminosae : Caesalpinioideae. Contribution à l'étude de l'histoire biogéographique des légumineuses. Ph.D. Thesis, Université de Montréal. 2006; 308 pp., Montréal.

Fougère-Danezan, M., Herendeen, P.S., Maumont, S., Bruneau, A. Morphological evolution in the variable resin-producing Detarieae (Fabaceae): do morphological characters retain a phylogenetic signal? Annals of Botany. 2010; 105:311–325.

Gaertner, J. De Fructibus et Seminibus Plantarum : accedunt seminum centuriae quinque priores cum tabulis. 1791; 2, 306 pp., Stutgardiae, Tubingae.

Gallieni, J.S. Neuf ans à Madagascar. Librairie Hachette et Co. 1908; 372 pp., Paris.

Ganzhorn, J.U., Lowryii, P.P., Schatz, G.E., Sommer, S. The biodiversity of Madagascar: one of the world's hottest hotspots on its way out. Oryx. 2001; 35:346–348.

Global Forest Watch (February 2019). https://www.globalforestwatch.org/dashboards/country/MDG

Grimalt, J., Simoneit, R.T., Hatcher, P.G., Nissenbaum, A. The molecular composition of ambers. Organic Geochemistry. 1988; 13:677–690.

Griswold, C., Wood, H., Carmichael, A. The lace web spiders (Araneae, Phyxelididae) of Madagascar: phylogeny, biogeography and taxonomy. Zoological Journal of the Linnean Society. 2012; 164:728–810.

Guiliano, M., Asia, L., Onoratini, G., Mille, G. Applications of diamond crystal ATR FTIR spectroscopy to the characterization of ambers. Spectrochimica Acta. 2007; part A 67, 1407–1411.

Guilbert, E., Heiss, E. First fossil records of Tingidae from Madagascan Copal with description of two new species (Hemiptera, Heteroptera). Linzer Biologische Beiträge. 2016; 48:1081–1090.

Gustafson, G.T., Prokin, A.A., Bukontaite, R., Bergsten, J., Miller, K.B. Tip-dated phylogeny of whirligig beetles reveals ancient lineage surviving on Madagascar. Scientific Reports. 2017; 7:8619, https://www.nature.com/articles/s41598-017-08403-1

Harms, D., Dunlop, J.A. The fossil history of pseudoscorpions (Arachnida: Pseudoscorpiones). Fossil Record. 2017; 20:215–238.

Hawlitschek, O., Ramírez Garrido, S., Glaw, F. How marine currents influenced the widespread natural overseas dispersal of reptiles in the Western Indian Ocean region. Journal of Biogeography. 2016; 44:1426–1440.

Heikkilä, M., Brown, J.W., Baixeras, J., Mey, W., Kozlov, M.V. Re-examining there are and the lost : a review of fossil Tortricidae (Lepidoptera). Zootaxa. 2018 ; 4394: 41–60.

Hervieu, J. Géographie des sols malgaches. Essai synthétique. Cahiers ORSTOM Pédologie V. 1967; 39–82.

Hills, E.S. Fossiliferous Tertiary resin from Allendale, Victoria. Proceedings of the Royal Society of Victoria. 1957; 69:15–19.

Horton, M.C. Shanga: The Archaeology of a Muslim Trading Community on the Coast of East Africa. British Institute in Eastern Africa. 1996; xvi + 458 pp., London.

Howes, F.N. Vegetable gums and resins. Chronica Botanica Company. 1949; 188 pp., Waltham, Massachusetts.

Iturralde-Vinent, M.A. Geology of the amber-bearing deposits of the Greater Antilles. Caribbean Journal of Science. 2001; 37: 141–167.

Jacob, G. La France et Madagascar de 1880 à 1894. Aux origines d’une conquête coloniale. RAHIA hors-série, Centre d’Étude des Mondes Africains (CEMAF) MMSH. 1996; 195 pp., Aix-en-Provence.

Jones, A., Breuning-Madsen, H., Brossard, M., Dampha, A., Deckers, J., Dewitte, O. et al. (Eds.) Soil Atlas of Africa. European Commission, Publications Office of the European Union. 2013; 176 pp., Luxembourg.

Kew Science. *Hymenaea verrucosa* Gaertn. http://powo.science.kew.org/taxon/urn:lsid:ipni.org:names:498815-1#distribution-map [last access November 2019].

Kimura, H., Tsukada, Y., Chujo, R., Sasaki, K. Structural study of amber by high-resolution solid-state NMR. Amber Reports. Society of Amber Studies of Japan. 2006a; 6:1–14 (in Japanese).

Kimura, H., Tsukada, Y., Mita, H., Yamamoto, Y., Chujo, R., Yukawa, T. A spectroscopic index for estimating the age of amber. Bull Chemical Society of Japan. 2006b; 79:451–453.

Kirk, J. Identification of the Modern Copal Tree, *Trachylobium Hornemannianum*, with that which yielded the Copal or Animi now found in the earth on the East Coast of Africa, often where no Copal-yielding trees now exist. Bot J Lin Soc. 1876; 15:234–235.

Klimke, J., Franke, D., Mahanjane, E.S., Leitchenkov, G. Tie-points for Gondwana reconstructions from a structural interpretation of the Mozambique Basin, East Africa, and the Riiser-Larsen Sea, Antarctica. Solid Earth Discuss. 2017; https://doi.org/10.5194/se-2017-80

Koch, H. Combining morphology and DNA barcoding resolves the taxonomy of Western Malagasy *Liotrigona* Moure, 1961 (Hymenoptera: Apidae: Meliponini. African Invertebrates. 2010; 51:413–421.

Kosmowska-Ceranowicz, B. Definitions and nomenclature of fossil resins. In: Kosmowska-Ceranowicz, B., Gierlowski, W. (Eds.), Amber Views, Opinions, The International Amber Association, Gdansk, 2006; pp. 75–77.

Kottek, M., Grieser, J., Beck, C., Rudolf, B., Rubel, F. World Map of the Köppen-Geiger climate classification updated. Meteorologische Zeitschrift. 2006; 15:259–263.

Krause, D.W., Sertich, J.J., O'Connor, P.M., Rogers, K.C., Rogers, R.R. The Mesozoic Biogeographic History of Gondwanan Terrestrial Vertebrates: Insights from Madagascar's Fossil Record. Annual Review of Earth and Planetary Sciences. 2019; 47:519–553.

Krishna, M.P., Mohan, M. Litter decomposition in forest ecosystems: a review. Energy, Ecology and Environment. 2017; 2:236–249.

Labandeira, C.C. Amber. In: Laflamme, M., Schiffbauer, J.D., Darroch, S.A.F. (Eds.), Reading and Writing of the Fossil Record: Preservational Pathways to Exceptional Fossilization. The Paleontological Society Papers 20, The Paleontological Society Short Course. 2014; pp. 163–217.

Lambert, J.B., Johnson, S.C., Poinar, G.O. Jr. Resin from Africa and South America: criteria for distinguishing between fossilized and recent resin based on NMR spectroscopy. In: Anderson, K.B., Crelling, J.C. (Eds.), Amber, Resinite, and Fossil Resins, American Chemical Society Symposium Series, No. 617, American Chemical Society, Washington, DC. 1995; pp. 193–202.

Lambert, J.B., Tsai, C.Y.-H, Shah, M.C., Hurtley, A.E., Santiago-Blay, J.A. Distinguishing amber and copal classes by Proton Magnetic Resonance Spectroscopy. Archaeometry. 2012; 54:332–348.

Langenheim, J.H. Biology of amber-producing trees: focus on case studies of *Hymenaea* and *Agathis*. In: Anderson, K.B., Crelling, J.C. (Eds.), Amber, Resinite, and Fossil Resins,

American Chemical Society Symposium Series, No. 617, American Chemical Society, Washington, DC. 1995; pp. 1–31.

Langenheim, J.H. Plant resins. Chemistry, Evolution, Ecology, Ethnobothany. Timber Press. 2003; 586 pp., Oregon.

Langenheim, J.H., Lee, Y.T. Reinstatement of the genus *Hymenaea* (Leguminosae: Caesalpinioideae) in Africa. Brittonia. 1974; 26:3–21.

Lee, Y.-T., Langenheim, J.H. Systematics of the genus *Hymenaea* L. Leguminosae, Caesalpinioideae. Detarieae. University of California Publication in Botany. 1975; 69:1–109.

Le Galle, Ph., Silvain, J.-F., Nel, A., Lachaise, D. Les insectes actuels témoins des passés de l'Afrique: essai sur l'origine et la singularité de l'entomofaune de la région afrotropicale. Annales de la Société entomologique de France, nouvelle série. 2010; 46:297–343.

Lewton, F.L. A Classification of Gums, Resins and Similar Substances. American Journal of Pharmacy. 1899; 71:270-274.

Lourenço, W.R. Premier cas connu d'un sub-fossile de scorpion dans le copal de Madagascar. Compte Rendus de l'Académie des Sciences. 1996; sér. II a:889–891.

Lourenço, W.R. Premier cas d'un sub-fossile d'araignée appartenant au genre *Archaea* Koch & Berendt (Archaeidae) dans le copal de Madagascar. Comptes Rendus de l'Académie des Sciences. 2000; sér. II a:509–512.

Lourenço, W.R. A new sub-fossil scorpion of the genus *Microcharmus* Lourenço from Malagasy copal (Scorpiones, Microcharmidae. Boletín Sociedad Entomólogica Aragonesa. 2009; 44:135–137.

Lourenço, W.R., Henderickx, H. Another new sub-fossil species of scorpion of the genus *Palaeogrosphus* Lourenço, 2000 from Malagasy copal (Scorpiones: Buthidae). Euscorpius. 2012; 137:1–4.

Lutjeharms, J.R.E., Bang, N.D., Duncan, C.P. Characteristics of the currents east and south of Madagascar. Deep Sea Research Part A. Oceanographic Research Papers. 1981; 28:879–899.

Martínez-Delclòs, X., Briggs, D.E.G., Peñalver, E. Taphonomy of insects in carbonates and amber. Palaeogeography, Palaeoclimatology, Palaeoecology. 2004; 203:19–64.

Massa, B. New taxa of Orthoptera (Insecta Tettigoniidae Phaneropterinae) from Madagascar. Zootaxa. 2017; 4242:299–312.

Maundrell, H. A Visit to the North-East Province of Madagascar. The Journal of the Royal Geographical Society of London. 1867; 37:108–116.

McCall, R. Implications of recent geological investigations of the Mozambique Channel for the mammalian colonization of Madagascar. Proceedings of the Royal Society B. 1997; 264:663–665.

McCoy, V.E., Boom, A., Solórzano Kraemer M.M., Gabbott, S.E. The chemistry of American and African amber, copal, and resin from the genus *Hymenaea*. Organic Geochemistry. 2017; 113:43–54.

McIntosh, J.G. The Manufacture of Varnishes and Kindred Industries based on and included the Drying Oils and Varnishes of Ach. Livache. Scott, Greenwood & Son. 1908; 209 pp., London.

McKey, D. Legumes and nitrogen: the evolutionary ecology of a nitrogen-demanding lifestyle. In: Sprent, J.I., McKey, D. (Eds.), Advances in Legume Systematics, Part 5. The Nitrogen Factor, Royal Botanic Gardens, Kew. 1994; pp. 211–228.

Meldola, R. The Forest Products of Madagascar. Journal of the Society of Arts. 1891; 39:787–798.

Meunier, F. Sur deux Mymarinae du Copal récent de Madagascar et de Zanzibar (Hymenoptera). Bulletin de la Société Entomologique de France. 1909; 14:145–149.

Meunier, F. Über einige Proctotrypidae (Bethylinae, Ceraphroninae und Scelioninae) aus dem subfossilen und rezenten Kopal von Zanzibar und von Madagaskar. Zeitschrift der Deutschen Geologischen Gesellschaft. 1916; 68:391–395.

Meyer, C., Todd, J.M., Beck, C.W. From Zanzibar to Zagros: a copal pendant from Eshnunna. Journal of Near Eastern Studies. 1991; 50:289–298.

Missouri Botanical Garden. *Catalogue of the Plants of Madagascar*. Tropicos.org. Missouri Botanical Garden. http://www.tropicos.org/Name/13020527?projectid=17 [last access July 2019].

Myers, N., Mittermeier, R.A., Mittermeier, C.G., da Fonseca, G.A.B., Kent, J. Biodiversity hotspots for conservation priorities. Nature. 2000; 403:853–858.

Oldeman, R.A.A. Dynamics in tropical rain forests. In: Holm-Nielsen, L.B., Nielsen, L.C., Balslev, H. (Eds.), Tropical forests - Botanical dynamics, speciation and diversity. Academic Press. 1990; pp. 3–21, London.

Oliver, D. Flora of Tropical Africa II. L. Reeve & Co. 1871; pp. 311–312, London.

Oliver, S.P. On the Hovas of Madagascar. Journal of the Anthropological Society of London. 1868; 6:119-125.

Penney, D. Sub/fossil resin research in the 21st Century: trends and perspectives. Paläontologische Zeitchrift. 2016; 90:425–447.

Peñalver, E., Delclòs, X., Solórzano-Kraemer, M.M. A New Approach to determine the resiniferous trees evolved in the origin of the Cretaceous amber deposits. 1st Palaeontological Virtual Congress, Palaeontology in the virtual era. Book of abstracts. 2018; ISBN: 978-84-09-07386-3, Valencia.

Peris, D., Solórzano Kraemer, M.M., Peñalver, E., Delclòs, X. New ambrosia beetles (Coleoptera: Curculionidae: Platypodinae) from Miocene Mexican and Dominican ambers and their paleobiogeographical implications. Organism Diversity & Evolution. 2015; 15:527–542.

Perrichot, V., Boudinot, B., Cole, J., Delhaye-Prat, V., Esnault, J. African fossiliferous amber: a review. 7th International conference on Fossil Insects, Arthropods, and Amber, Abstracts 41. 2016; Edinburgh.

Perrichot, V., Boudinot, B., Chény, C., Cole, J., Jeanneau, L., Schmidt, A. et al. The age and paleobiota of Ethiopian amber revisited. 5th International Paleontological Congress, Abstracts 24. 2018; Paris.

Poinar, G.O. Jr. Life in Amber, Stanford University Press. 1992; 368 pp., Stanford.

Poinar, G.O. Jr. Cenozoic fauna and flora in amber. Estudios del Museo Ciencias Naturales de Álava. 1999; 14 (Número Especial 2):151–154.

Poinar, G., Brow, A., Brown, S., Poinar, R. Stuck in Time (Madagascar copal). Fauna magazine. 2001; 2:70–76.

Pollard, E., Kinyera, O.Ch. 2017. The Swahili Coast and the Indian Ocean Trade Patterns in the 7th–10th Centuries CE. Journal of Southern African Studies. 2017; 43:927–947.

Powers, J.S., Montgomery R.A., Adair, E.C., Brearley, F.Q., DeWalt, S.J. et al. Decomposition in tropical forests: a pan-tropical study of the effects of litter type, litter placement and mesofaunal exclusion across a precipitation gradient. Journal of Ecology. 2009; 97:801–811.

Ragazzi, E., Roghi, G., Giaretta, A., Gianolla, P. Classification of amber based on thermal analysis. Thermochimica Acta. 2003; 404:43–54.

Rakotovao Andrianavah, M. Carte Paléontologique de Madagascar. Inventaire et mise en valeur du patrimoine paléontologique. Thèse de Doctorat, Université de Toulousse. 2015; 448 pp., Toulousse.

Ramifehiarivo, N., Brossard, M., Grinand, C., Andriamananjara, A., Razafimbelo, T., Rasolohery, A. et al. Mapping soil organic carbon on a national scale: Towards an improved and updated map of Madagascar. Geoderma Regional. 2017; 9:29–38.

Razanamalala, K., Razafimbelo, T., Maron, P.-A., Ranjard, L., Chemidlin, N., Lelièvre, M. et al. Soil microbial diversity drives the priming effect along climate gradients: a case study in Madagascar. The ISME Journal. 2017; 12:451–462.

Regert, M., Devièse, T., Le Hô, A.-S., Rougeulle, A. Reconstructing ancient Yemeni commercial routes during the middle ages using structural characterization of terpenoid resins. Archaeometry. 2008; 50:668–695.

Retallack, G.J., Wynn, J.G., Benefit, B.R., McCrossin, M.L. Paleosols and paleoenvironments of the Middle Miocene, Maboko Formation, Kenya. Journal of Human Evolution. 2002; 42:659–703.

Rice, P. Amber: Golden Gem of the Ages. AuthorHouse Publ., 2006; 321 pp., Bloomington, Indiana.

Riquelme, F., Villegas-Guzmán, G., González-Santillán, E., Córdova-Tabares, V., Francke, O.F., Piedra-Jiménez, D. et al. New Fossil Scorpion from the Chiapas Amber Lagerstätte. PLOS ONE. 2015; 10(8):e0133396.

Riquier, J. Définition et classification des sols ferrallitiques de Madagascar. Cahiers ORSTOM Pédologie. 1966; 4:75–89.

Roederer, P. Les sols de Madagascar. Cahiers ORSTOM Pédologie. 1971; 5:1–56.

Samonds, K.E., Godfrey, L.R., Alic, J.R., Goodmand, S.M., Vences, M., Sutherland, M.R. et al. Spatial and temporal arrival patterns of Madagascar's vertebrate fauna explained by distance, ocean currents, and ancestor type. Proceedings of the National Academy of Sciences USA. 2012; 109:5352–5357.

Sanchez, S. État marchand et État agraire dans l'océan Indien occidental: le sultanat de Zanzibar et le royaume de Madagascar (1817–1874). Cahiers d'histoire. Revue d'histoire critique. 2015; 128:37–57.

Schlüter, D., Glöckner, W. Bernstein – Bernsteine und Bernstein-Fossilien. Stuttgarter Beiträge zur Naturkunde ser. C. 1978; 8:1–72.

Schlüter, T., von Gnielinski, F. The East African Copal: Its Geologic, Stratigraphic, Palaeontologic Significance and Comparison with Other Fossil Resins of Similar Age. Occasional Paper, Natural Museums of Tanzania. 1986; 8:1–32.

Schmidt, A.R., Perrichot, V., Svojtka, M., Anderson, K.B., Belete, K.H., Bussert, R. et al. Cretaceous African life captured in amber. Proceedings of the National Academy of Sciences USA. 2010; 107:7329–7334.

Schrire, B.D., Lavin, M., Lewis, G.P. Global distribution patterns of the Leguminosae: insights from recent phylogenies. In: Friis, I., Balslev, H. (Eds.), Plant Diversity and Complexity Patterns - Local, Regional and Global Dimensions. Biologiske Skrifter. 2005; 55:375–422.

Solórzano Kraemer, M.M. Systematic, palaeoecology, and palaeobiogeography of the insect fauna from Mexican amber. Palaeontographica Abteilung A. 2007; 1–133.

Solórzano Kraemer, M.M., Delclòs, X., Clapham, M.E., Arillo, A., Peris, D., Jäger, P. et al. Arthropods in modern resins reveal if amber accurately recorded forest arthropod communities. Proceedings of the National Academy of Sciences USA. 2018; 115:6739–6744 + supplementary material.

Solórzano Kraemer, M.M., Kraemer, A.S., Stebner, F., Bickel, D.J., Rust, J. Entrapment Bias of Arthropods in Miocene Amber Revealed by Trapping Experiments in a Tropical Forest in Chiapas, Mexico. PLOS ONE. 2015; 10(5):e0126046.

Speranza, M., Ascaso, C., Delclòs, X., Peñalver, E. Cretaceous mycelia preserving fungal polysaccharides: taphonomic and paleoecological potential of microorganisms preserved in fossil resins. Geologica Acta. 2015; 13:363–385.

Springer, M.S., Meredith, R.W., Janecka, J.E., Murphy, W.J. The historical biogeography of Mammalia. Phil Trans Roy Soc B. 2011; 366:2478–2502.

Stach, P., Martinkutė, G., Šinkūnas, P., Natkaniec-Nowak, L., Drzewicz, P., Naglik, B. et al. An attempt to correlate the physical properties of fossil and subfossil resins with their age and geographical location. Journal of Polymer Engineering. 2019; 39:1–13.

Sunseri, Th. The political ecology of the copal trade in the Tanzanian coastal hinterland, c. 1820–1905. Journal of African History. 2007; 48:201–220.

Tantely, M.L., Le Goff, G., Boyer, S., Fontenille, D. An updated checklist of mosquito species (Diptera: Culicidae) from Madagascar. Parasite. 2016; 23:20 (https://www.ncbi.nlm.nih.gov/pmc/articles/PMC4840257/)

Tibell, L., Titov, A. Species of *Chaenothecopsis* and *Mycocalicium* (Caliciales) on exudate. The Bryologist. 1995; 98:550–560.

Tower Gallery Natural Copal Madagascar selected from the TowerCrystals internet collection. http://towercrystals.com/amber/madagascar/copal/index.html [last access February 2019].

Vankerkhoven, F., Henderickx, H., Dekoninck, W. *Anochetus madagascarensis* Forel, 1887 found in Madagascan copal (Hymenoptera: Formicidae). Société royale belge d'Entomologie / Koninklijke Belgische Vereniging voor Entomologie. 2010; 146:216–219.

Vávra, N. Amber, fossil resins, and copal—contributions to the terminology of fossil plant resins. Denisia. 2009; 86:213–222.

Vitali, F. About two *Batrachorhina*-species included in Malagasy copal (Coleoptera, Cerambycidae). Entomapeiron Paleoentomology. 2006; 1:13–28.

Vitali, F. About some sub-fossil Glaucytini included in Malagasy copal (Coleoptera, Cerambycidae). Entomapeiron Paleoentomology. 2007; 2:7–13.

White, F. Long distance dispersal, overland migration and extinction in the shaping of tropical African floras. Bothalia. 1983; 14:395–403.

Wichura, H., Bousquet, R., Oberhänsli, R., Strecker, M.R., Trauth, M.H. 2011. The Mid-Miocene East African Plateau: a pre-rift topographic model inferred from the emplacement of the phonolitic Yatta lava flow, Kenya. Geological Society Special Publications. 2011; 357:285–300.

Williamson, G.C. The Book of Amber. Ernest Benn Limited. 1932; xiii + [i] + 15–268 pp., London.

Figure 1

Click here to download Figure Figure 1.pdf

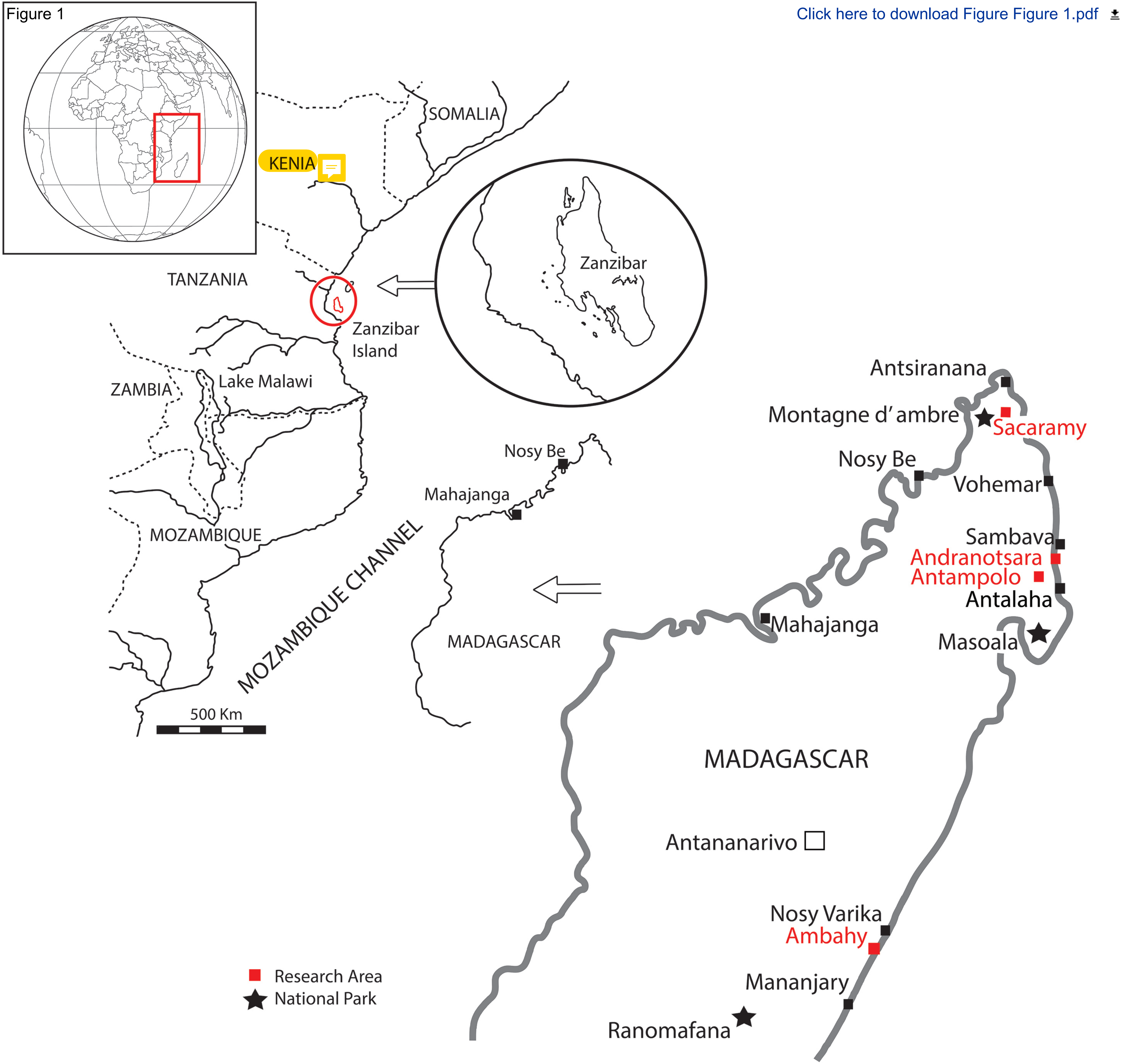

Figure 2

Click here to download Figure Figure 2.pdf

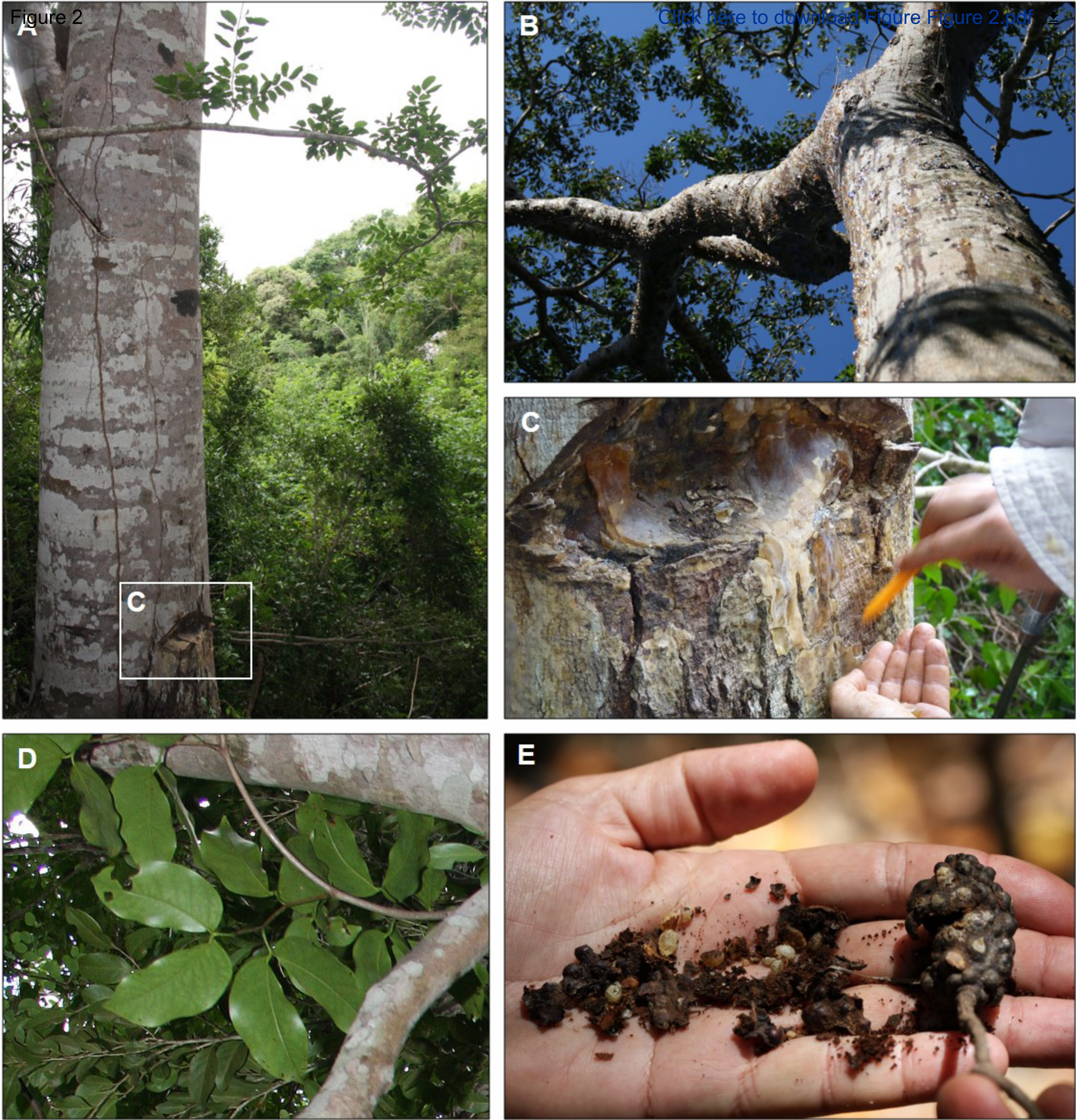

Figure 3

Click here to download Figure Figure 3_1.pdf

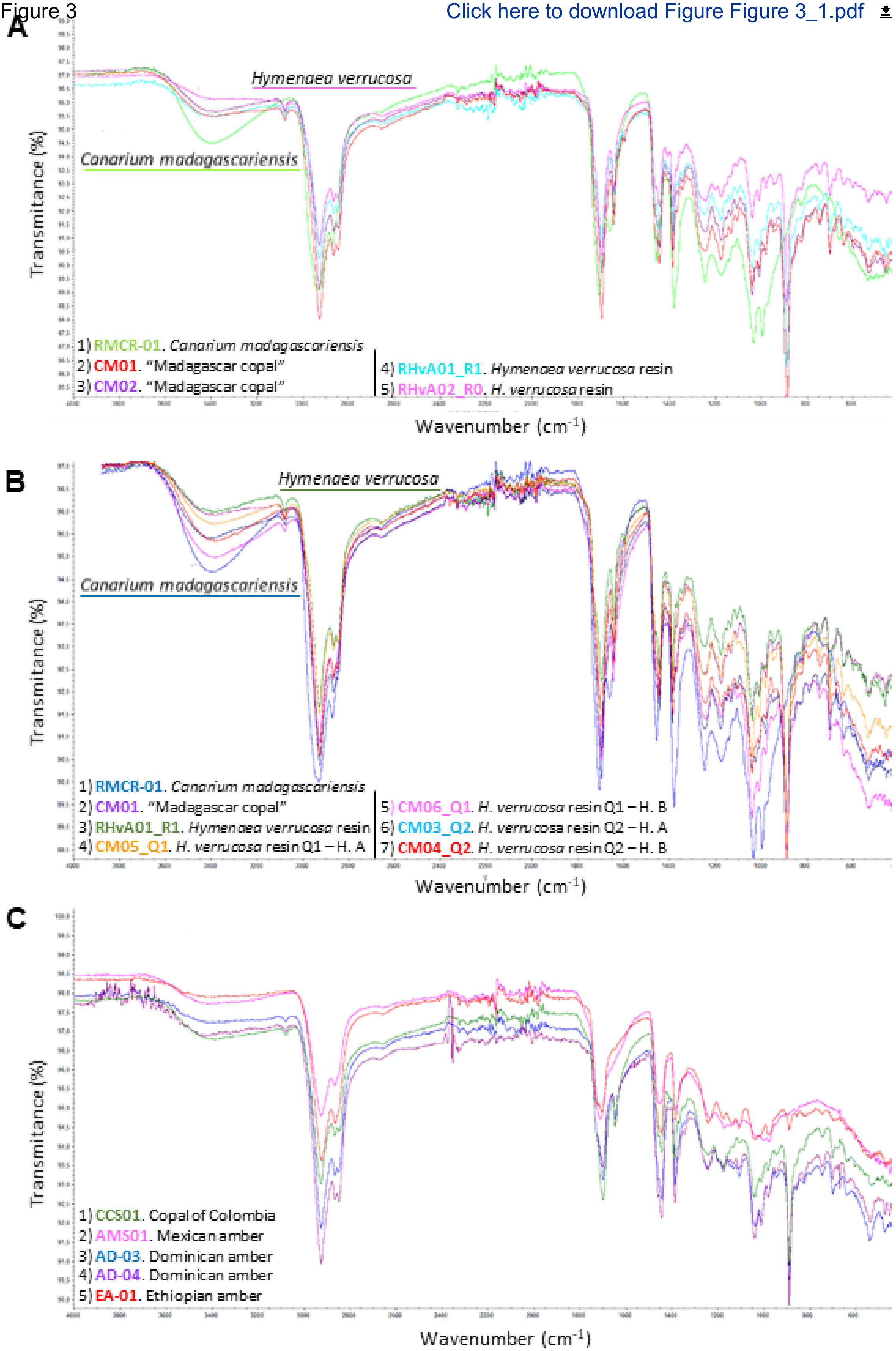

Figure 4

Click here to download Figure Figure 4.pdf

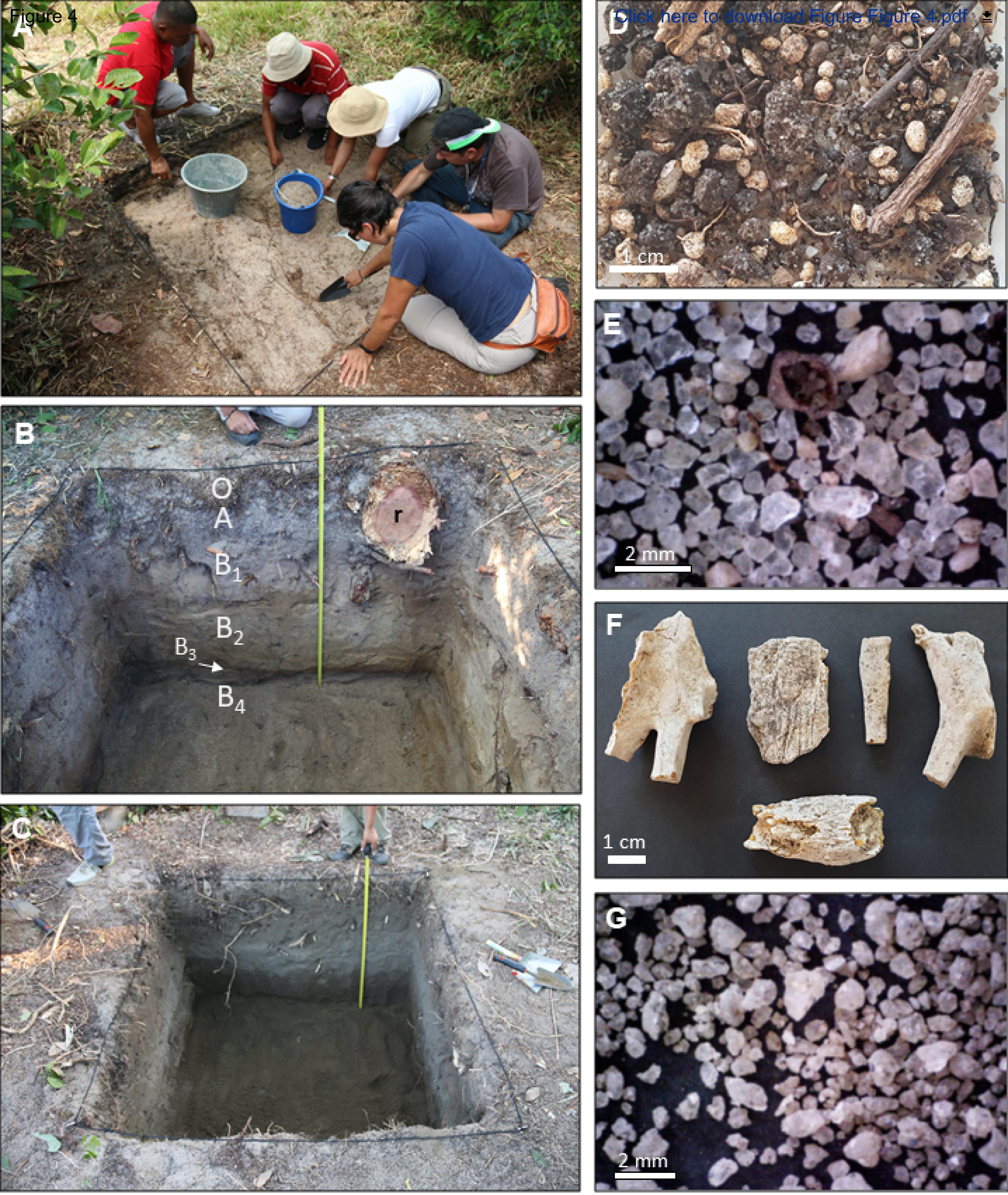

Figure 5 Click here to download Figure Figure 5.pdf

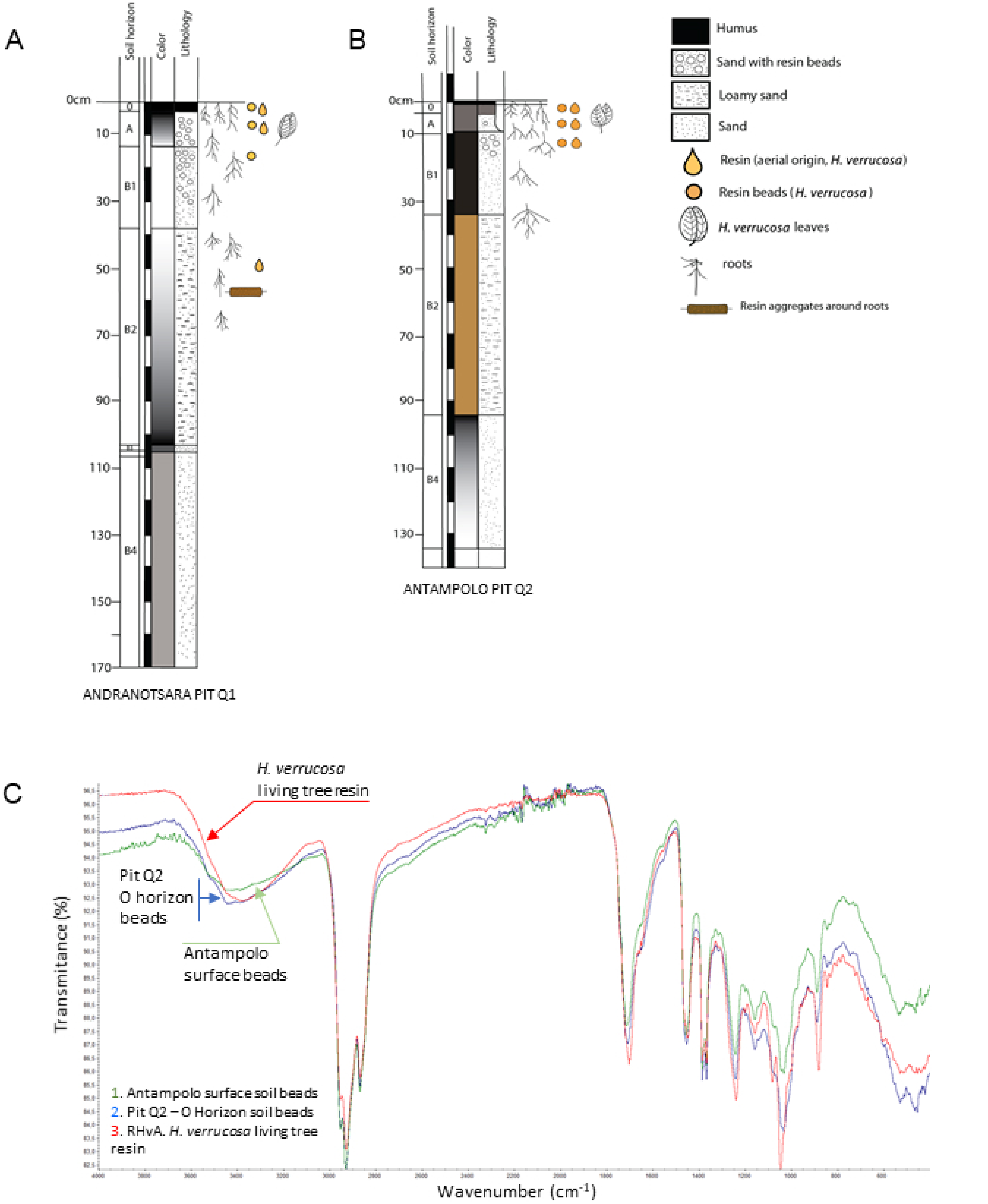

Figure 6

Click here to download Figure Figure 6.pdf

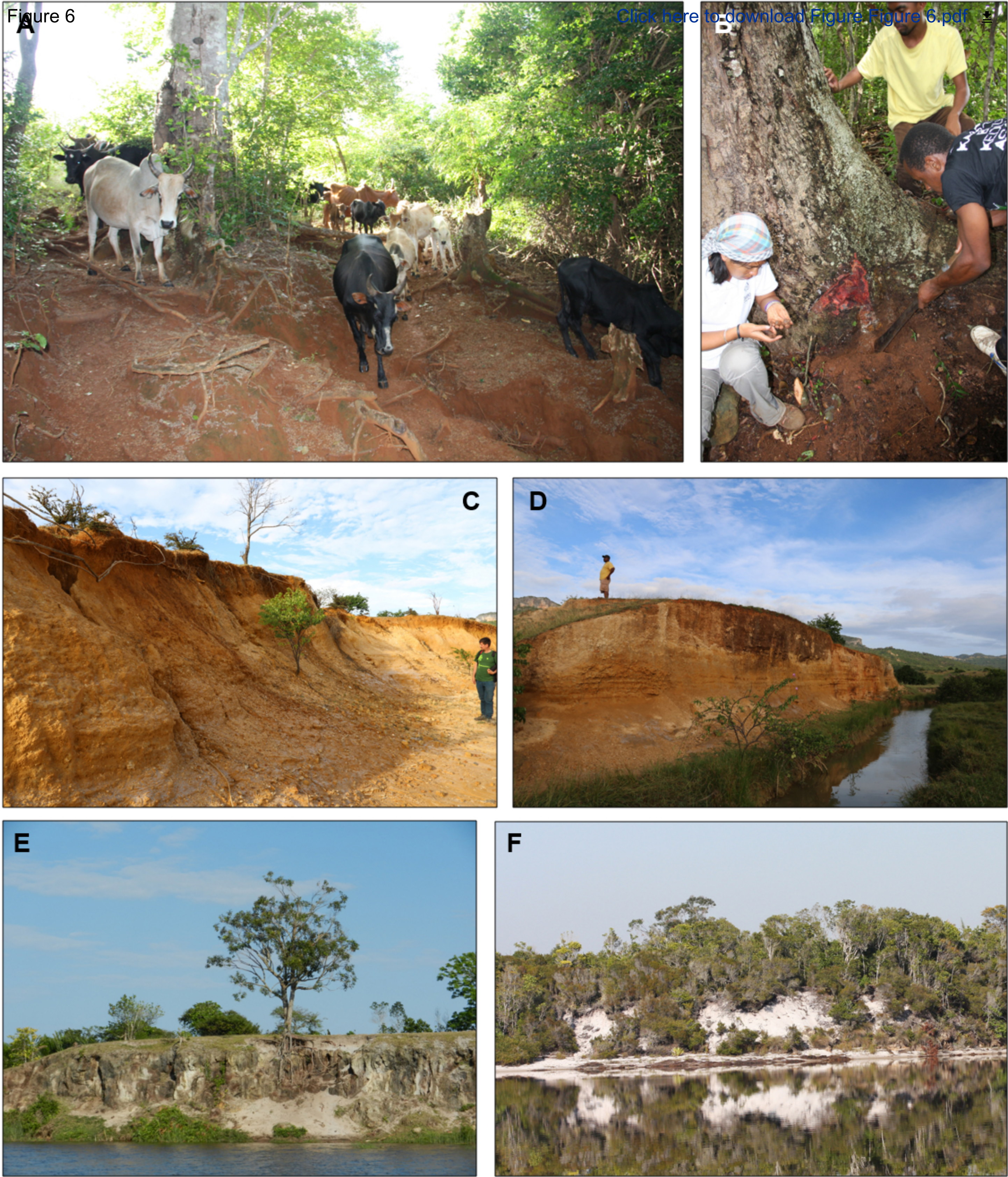

Figure 7

Click here to download Figure Figure 7_1.pdf

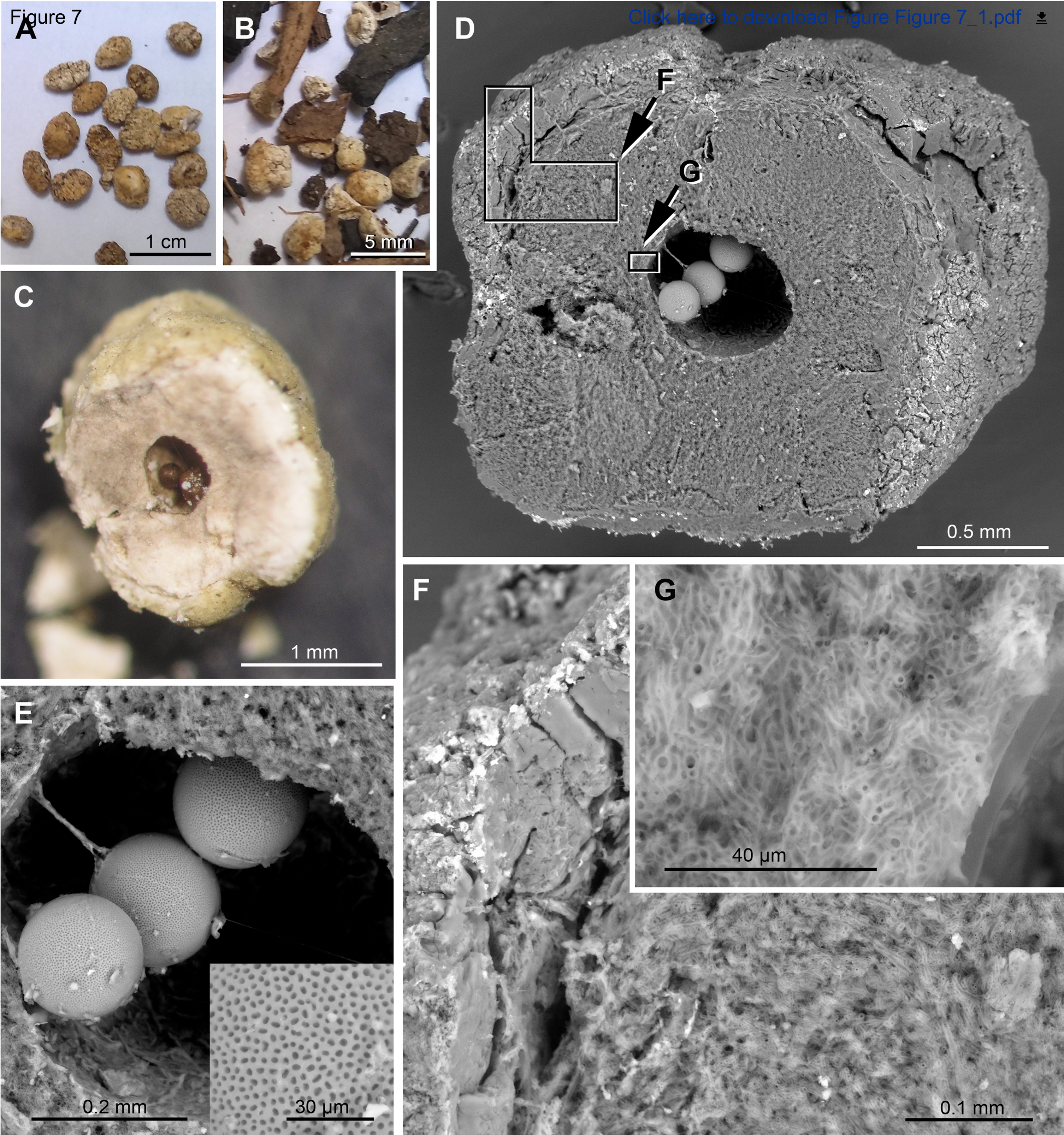

Figure 8 Click here to download Figure Figure 8.pdf

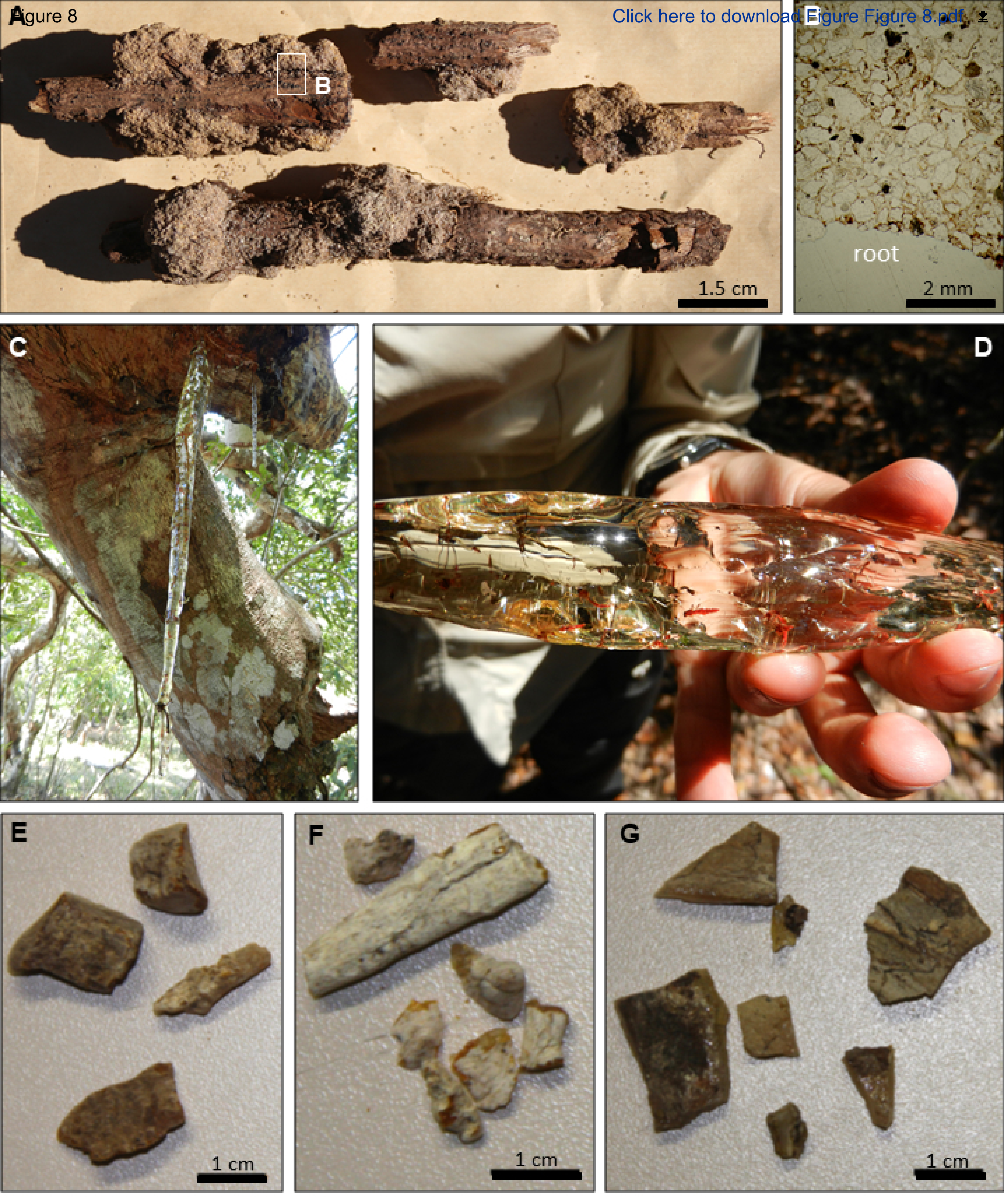

Supplement: Attachment — Submitted filename: PONE-D-19-32304_reviewer1.pdf [file pone.0232623.s003.pdf]
