# Supplementary material for: Unravelling the mystery of “Madagascar copal”: Age, origin and preservation of a Recent resin
Source: PLoS One. 2020 May 18;15(5):e0232623. doi: 10.1371/journal.pone.0232623 (PMC7233546; doi:10.1371/journal.pone.0232623)
Supplement: Attachment — Submitted filename: Response to reviewers 3.pdf [file pone.0232623.s005.pdf]

ANSWER TO REVIEWERS

EDITOR

Additionally, I have one comment as a follow-up to the authors' response:

Line 843: the term primitive here is in fact imprecise in a phylogenetic context. I recommend stating "species sister to all remaining Hymenaea species". Please see this publication for a discussion on "primitive" in phylogenetics: Omland, K.E., Cook, L.G. and Crisp, M.D., 2008. Tree thinking for all biology: the problem with reading phylogenies as ladders of progress. BioEssays, 30(9), pp.854-867.

Thanks for suggesting this article to us; it is very well written and clarifies some concepts. In general, we work in phylogenetic analysis, so we are familiar with the term's "sister" and "primitive", but the article really makes us think about how to interpret some phylogenetic hypotheses. Based on some phylogenetic proposals (Langenheim and Lee, 1974 or Fougère-Danezan, 2006) we included in the MS that Hymenaea verrucosa *was "the most primitive species of* Hymenaea*, and from which all the Neotropical species derived".* Hymenaea verrucosa *was considered primitive compared to the other species of the genus by Langenheim and Lee, who proposed the first phylogenetic hypotheses of the genus, based on the structure of the flowers. It was Fougère-Danezan who proposed* H. verrucosa *as a sister to all the other species of* Hymenaea*, with Neotropical distribution. After reading the proposed paper, we consider that perhaps our text was not accurate.*

However, the new proposal by De la Estrella et al. (2018) proposes H. verrucosa *as a sister to* H. oblongifolia *from northern South America and these two as sister to all other species of the genus. This new phylogenetical proposal does not affect our conclusions. Since the MS is not concerned with the* Hymenaea *phylogeny and evaluating the different proposals, we prefer, if you agree, to delete this sentence in the final version.*

- De la Estrella, M., Forest, F., Klitgård, B., Lewis, G.P., Mackinder, B.A., de Queiro, D.P., Wieringa, J.J., Bruneau, A. A new phylogeny-based tribal classification of subfamily Detarioideae, an early branching clade of florally diverse tropical arborescent legumes. Scientific Reports. 2018; 8:6884.
- Fougère-Danezan, M., Herendeen, P.S., Maumont, S., Bruneau, A. Morphological evolution in the variable resin-producing Detarieae (Fabaceae): do morphological characters retain a phylogenetic signal? Annals of Botany. 2010; 105:311–325.
- Langenheim, J.H., Lee, Y.T. Reinstatement of the genus *Hymenaea* (Leguminosae: Caesalpinioideae) in Africa. Brittonia. 1974; 26:3–21.

Comments to the Author

1. If the authors have adequately addressed your comments raised in a previous round of review and you feel that this manuscript is now acceptable for publication, you may indicate that here to bypass the “Comments to the Author” section, enter your conflict of interest statement in the “Confidential to Editor” section, and submit your "Accept" recommendation.

Reviewer #1: (No Response)

Reviewer #2: All comments have been addressed

2. Is the manuscript technically sound, and do the data support the conclusions?

The manuscript must describe a technically sound piece of scientific research with data that supports the conclusions. Experiments must have been conducted rigorously, with appropriate controls, replication, and sample sizes. The conclusions must be drawn appropriately based on the data presented.

Reviewer #1: Yes

Reviewer #2: Yes

3. Has the statistical analysis been performed appropriately and rigorously?

Reviewer #1: N/A

Reviewer #2: N/A

4. Have the authors made all data underlying the findings in their manuscript fully available?

The PLOS Data policy requires authors to make all data underlying the findings described in their manuscript fully available without restriction, with rare exception (please refer to the Data Availability Statement in the manuscript PDF file). The data should be provided as part of the manuscript or its supporting information, or deposited to a public repository. For example, in addition to summary statistics, the data points behind means, medians and variance measures should be available. If there are restrictions on publicly sharing data—e.g. participant privacy or use of data from a third party—those must be specified.

Reviewer #1: Yes

Reviewer #2: Yes

5. Is the manuscript presented in an intelligible fashion and written in standard English?

PLOS ONE does not copyedit accepted manuscripts, so the language in submitted articles must be clear, correct, and unambiguous. Any typographical or grammatical errors should be corrected at revision, so please note any specific errors here.

Reviewer #1: Yes

Reviewer #2: Yes

6. Review Comments to the Author

Please use the space provided to explain your answers to the questions above. You may also include additional comments for the author, including concerns about dual publication, research ethics, or publication ethics. (Please upload your review as an attachment if it exceeds 20,000 characters)

Reviewer #1:

I am pleased that the authors considered my title suggestion and at least some of my other concerns.

I recommended citing Seyfullah et al. (2018) as is not exclusively a review paper but as it also reports many new observations. It discusses and illustrates preservation versus degradation of resins in soils of humid tropical forest (including observed time of complete resin degradation), also considering the relevance of resinicolous fungi. Refer, for example, to the 'Formation of amber deposits' chapter, tracing the path from resin exudation to the deposit. See also fig. 13. where resin on and inside forest soils is shown from the Pacific region and fig. 8 for resin preserved in peat swamps. The paragraphs on pages 1705-1706 have thus direct relation to the current study. If this paper is not worth citing here, the authors could delete approximately 30% of the presently cited literature from the manuscript.

Obviously, we totally disagree with the reviewer's last comment. In the first revision we did not cited some references proposed by this reviewer because all the observations make by their authors are of resins produced by gymnosperms. It is well known that the evolution of soils under angiosperm and gymnosperm forests are different, due to the rapid and slow degradation of their OM, respectively.

Nevertheless, because all the observations made by Seyfullah et al. (2008) were of the soil floor and leaf-litter, and because the climate are similar in our area and in the area studied by Seyfullah and collaborators, we would expect that the degradation and possible disappearance of the resin bodies are similar. For this reason, we include in the MS a brief new paragraph explaining that and introducing the research by Seyfullah et al. (2008).

I recommended citing the literature on resinicolous fungi and bacteria because the authors have randomly chosen the rather old study by Tibell & Titov (1995) which is important but not necessarily relevant for the current study. Speranza et al. (2015) studied supposed fungi from Cretaceous amber from Spain and (like Seyfullah et al. 2018, which is not considered by the authors to have any relation to the current study) from modern resin of New Zealand. Some of the results provided by Speranza et al. (2015) turned out to be controversial and have been revised by Saint Martin & Saint Martin (2017). Thus, the reasoning of the authors for excluding these references is not comprehensible.

We introduced the reference of Tibell & Titov (1995) because they explained the presence of resiniferous fungi on angiosperm exudates. It is true that we introduced our publication (Speranza et al. 2015) because some resiniferous Cretaceous fungi are discovered affecting amber pieces of Spain (araucariacean origin), and it is also true that some results turned out to be controversial for some other researchers, but because our research in Madagascar is only on angiosperm resins, we prefer do not cite both references (Speranza et al., 2015 and Saint Martin and Saint Martin, 2017). However, we include the reference Rikkinen & Schmidt (2018) because they explained the presence of calicioid lichens and fungi on the angiosperm exudates.

I recommended citing Rikkinen & Schmidt (2018) because this is the most recent ecological study on fossil and modern resinicolous fungi with reference to their relevance for interpretation of the forest ecosystems, as calicioid fungi serve as indicator taxa for modern old-growth forests. I recommended citing Beimforde et al. (2017) because it is likewise a direct observation of resinicolous fungi (and their interactions with insects) in a modern tropical forest ecosystem dominated by very resinous plants.

We consider that the inclusion of the reference Beimforde et al. (2017) is not suitable in the manuscript because that research is not related with our case study. These authors explained that the resin production of Araucaria humboldtensis *is closely related to the borer activity of the larvae of two different group of beetles. One species of ascomycete and diverse fungi growth in the surface of these gymnosperm exudates. The strong host specificity of one of the beetles, along with the occurrence of two exclusive fungi, makes the authors suggest that the resin associated community is native and has evolved on the endemic conifer host.*

line 446: 'The coastal forest harbors a very unusual arthropod community' – My previous question was: In what manner is it unique?
Thanks for explaining it to me, but the authors should likewise explain it to the readers.

We are included a paragraph explaining why.

Reviewer #2:

The comments and suggestions of both reviewers have been taken into account. I feel that the manuscript has been improved and gained significantly in clarity. Therefore, I recommend the acceptance.

Although I haven't specifically proofread the manuscript for spelling errors, I noticed a few things:

Line 57: write "detail" instead of "detain"
Line 81: "hinders" instead of "hiders"
Line 113: "one-million-year-old" instead of "one-million-years-old"
Line 271: add a space = "between 10,000" instead of "between10,000"
Line 478: "lasts" instead of "last"
Line 533 (figure caption): add a space = "from: 1." instead of "from:1."
Line 587 (figure caption): replace "Montage" by "Montagne"
Line 599: italicize the entire species name
Lines 684/685: Incorrect phrasing. Did you mean: "As explained above, we followed the proposal of Anderson (1996) suggesting a scale based on 14C dating"?
Line 737: replace "through" by "throughout"
Line 743: delete one "that" ("that lead" instead of "that that lead")
Besides, it doesn't look like the Table 1 is called in the text. Maybe it should be called at line 706? The sentence "The samples radiocarbon dated are the following:" is not followed by anything. A new paragraph starts after.

All the spelling errors observed by the reviewer 2 have been corrected.
