# Supplementary material for: Unravelling the mystery of “Madagascar copal”: Age, origin and preservation of a Recent resin
Source: PLoS One. 2020 May 18;15(5):e0232623. doi: 10.1371/journal.pone.0232623 (PMC7233546; doi:10.1371/journal.pone.0232623)
Supplement: Attachment — Submitted filename: Response to reviewers.docx [file pone.0232623.s004.docx]

| **Date:** | Jan 08 2020 05:21AM |
| --- | --- |
| **To:** | "Xavier Delclos" xdelclos@ub.edu |
| **From:** | "PLOS ONE" plosone@plos.org |
| **Subject:** | PLOS ONE Decision: Revision required [PONE-D-19-32304] |
| **Attachment(s):** | [PONE-D-19-32304_reviewer1.pdf](https://www.editorialmanager.com/pone/download.aspx?guid=%7bf42c42c3-9da7-4b51-8ed5-6dfe9c912a36%7d&scheme=13&id=394791) |

PONE-D-19-32304
"Madagascar copal": a new approach to study the loss of biodiversity during the Anthropocene

**Dear Prof Barden,**

**Next, we answer to the proposed changes, and suggestions for modification by the reviewers to the manuscript (in blue).**

Line 90: The scientific term “copal” is a problem, – ambiguous - changed

Line 141: mainly in the oriental area of – use "Eastern" - changed

Line 167 to 200: in my view, there is value in keeping elements of the history of trade. This recounting may help those with "Madagascar" or "Zanzibar Copal" in collections. However, as both reviewers mention, it would be useful to trim text where available. –

This chapter has been not deleted but its length has been reduced.

Line 713: Remove first "The" - done

Line 880: Might you mean "oldest" or "ancestral" instead of "most primitive"? The term primitive is imprecise in most contexts.

Most primitive is not imprecise in this case because we refer to *Hymenaeae verrucosa*, that is considered in all phylogenetic analyses’ sister of all other 14 species of the genus.

Please include the following items when submitting your revised manuscript:

- A rebuttal letter that responds to each point raised by the academic editor and reviewer(s). This letter should be uploaded as separate file and labeled 'Response to Reviewers'. Done
- A marked-up copy of your manuscript that highlights changes made to the original version. This file should be uploaded as separate file and labeled 'Revised Manuscript with Track Changes'. Done
- An unmarked version of your revised paper without tracked changes. This file should be uploaded as separate file and labeled 'Manuscript'. Done

Please note while forming your response, if your article is accepted, you may have the opportunity to make the peer review history publicly available. The record will include editor decision letters (with reviews) and your responses to reviewer comments. If eligible, we will contact you to opt in or out.

We wish to make the peer review history publicly available to possible readers.

Journal Requirements:

When submitting your revision, we need you to address these additional requirements:

1. Please ensure that your manuscript meets PLOS ONE's style requirements, including those for file naming. The PLOS ONE style templates can be found at http://www.plosone.org/attachments/PLOSOne_formatting_sample_main_body.pdf and <http://www.plosone.org/attachments/PLOSOne_formatting_sample_title_authors_affiliations.pdf>

Done.

2. In your manuscript, please provide additional information regarding the specimens used in your study. Ensure that you have reported specimen numbers and complete repository information, including museum name and geographic location. Done.

If permits were required, please ensure that you have provided details for all permits that were obtained, including the full name of the issuing authority, and add the following statement:

'All necessary permits were obtained for the described study, which complied with all relevant regulations.' Done, all permits requested and granted are included in the acknowledgments section.

If no permits were required, please include the following statement: 'No permits were required for the described study, which complied with all relevant regulations.'

For more information on PLOS ONE's requirements for paleontology and archaeology research, see https://journals.plos.org/plosone/s/submission-guidelines#loc-paleontology-and-archaeology-research.

3. We note that Figures 2A and 6B include images of participants in the study.

We preferred to change the images using other in which no face is observed, because we cannot contact the people in the Figures 2A and 6B.

If you are unable to obtain consent from the subject of the photograph, you will need to remove the figure and any other textual identifying information or case descriptions for this individual.

Figures changed.

4. Thank you for stating the following in the Acknowledgments Section of your manuscript:

*"We thank the Spanish Ministry of Economy, Industry and Competitiveness (Project CGL2014-52163), the Spanish Government (Project AEI/FEDER, UE CGL2017-84419), the National Geographic Global Exploration Fund Northern Europe (Project GEFNE 127-14), and the German VolkswagenStiftung (Project N. 90946), for financial support."*

We note that you have provided funding information that is not currently declared in your Funding Statement. However, funding information should not appear in the Acknowledgments section or other areas of your manuscript. We will only publish funding information present in the Funding Statement section of the online submission form.

Please remove any funding-related text from the manuscript and let us know how you would like to update your Funding Statement. Currently, your Funding Statement reads as follows:

"The funders had no role in study design, data collection and analysis, decision to publish, or preparation of the manuscript."

Done. Funding information has been removed from the text. It is now included in the Funding Statement section on the online submission form.

5. PLOS requires an ORCID iD for the corresponding author in Editorial Manager on papers submitted after December 6th, 2016. Please ensure that you have an ORCID iD and that it is validated in Editorial Manager. To do this, go to ‘Update my Information’ (in the upper left-hand corner of the main menu), and click on the Fetch/Validate link next to the ORCID field. This will take you to the ORCID site and allow you to create a new iD or authenticate a pre-existing iD in Editorial Manager. Please see the following video for instructions on linking an ORCID iD to your Editorial Manager account: <https://www.youtube.com/watch?v=_xcclfuvtxQ>

Done

6. We note you have included a table to which you do not refer in the text of your manuscript. Please ensure that you refer to Table 1 in your text; if accepted, production will need this reference to link the reader to the Table.

The two tables included in the manuscript are now cited in it.

**Reviewers' comments:**

Reviewer's Responses to Questions

**Comments to the Author**

1. Is the manuscript technically sound, and do the data support the conclusions?

The manuscript must describe a technically sound piece of scientific research with data that supports the conclusions. Experiments must have been conducted rigorously, with appropriate controls, replication, and sample sizes. The conclusions must be drawn appropriately based on the data presented.

Reviewer #1: Yes

Reviewer #2: Yes

2. Has the statistical analysis been performed appropriately and rigorously?

Reviewer #1: N/A

Reviewer #2: N/A

 3. Have the authors made all data underlying the findings in their manuscript fully available?

The [PLOS Data policy](http://www.plosone.org/static/policies.action#sharing) requires authors to make all data underlying the findings described in their manuscript fully available without restriction, with rare exception (please refer to the Data Availability Statement in the manuscript PDF file). The data should be provided as part of the manuscript or its supporting information, or deposited to a public repository. For example, in addition to summary statistics, the data points behind means, medians and variance measures should be available. If there are restrictions on publicly sharing data—e.g. participant privacy or use of data from a third party—those must be specified.

Reviewer #1: Yes

Reviewer #2: Yes

 4. Is the manuscript presented in an intelligible fashion and written in standard English?

PLOS ONE does not copyedit accepted manuscripts, so the language in submitted articles must be clear, correct, and unambiguous. Any typographical or grammatical errors should be corrected at revision, so please note any specific errors here.

Reviewer #1: No

Reviewer #2: Yes

We thank the two reviewers and the Editor for the effort in improving the English text. The current revised manuscript has been reviewed by the translation service of the University of Barcelona to improve the English.

5. Review Comments to the Author


**Reviewer #1:**

1) The paper is rather extensive, and, for convenience of the readership, all chapters can be shortened without any loss of essential information. The content of some paragraphs could be better be presented as tables to make the data better accessible for the audience. An example is the description of the FTIR data at p. 15, lines 348 – 371.

Some chapters have been shortened and now FTIR data are presented as a Table (Table 1)

2) The title does not reflect the content of the manuscript as the authors do not show a new approach for studying the biodiversity loss of Madagascar during the Anthropocene. A title like ‘Unravelling the mystery of ‘Madagascar copal’: age, origin and preservation of a rather young tree resin’ would much better fit to what is written. It is hard to extract the central theme. The authors should make clear what the actual focus of the study is.

The title of the manuscript has been changed as suggested this reviewer.

3) Lines 237 – 269 o the palaeobiogeography of *Hymenaea*, for example, are not at all relevant here.

We consider of high relevance the indication in this manuscript that the genus *Hymenaea* in Madagascar is present since the Miocene, and not latter, and that the lack of amber deposits from *Hymenaea* in Madagascar contrast to the rich Miocene amber deposits from Mexico or Dominican Republic. This circumstance only can be explained as different geological and environmental conditions. We preferred to maintain the original text.

4) Lines 694 – 710, as currently written, appear as pure speculation.

The speculative sentences about the possible origin of the beads in fungal infection and possible relationship of the fungus and the tree have been deleted. The other part of this section has been changed and now there is indication that the statements it contains are based on our taphonomic observations during the pit excavations and on the soil surfaces around.

5) Many actualistic observations on tree resins in modern forest ecosystems have recently been summarized by Seyfullah et al. 2018 (Biological Reviews). I wonder why this review paper has not been cited.

Our work analysed specifically the age of the copal from Madagascar. Seyfullah et al. (2018) is a very nice summary about the state-of-the-art of investigations about resin production, amber deposits, and amber and resin classification done during the last years but in our opinion, it is not relevant for the content of our manuscript.

6) lines 20-21, ‘In this regard, the study of ancient organisms, like those included in "Madagascar copal", is of relevance.’ Why ancient? The major result of the study is that they are modern.

We deleted the word “ancient”. In this context, the age of the resin is not relevant. Is important to mention that the study of the inclusions in Madagascar resin is important because “Madagascar copal” can be about 300 years old. In a region with a very high rate of biodiversity loss, such as the lowland coastal forest of the eastern Madagascar, due to intense anthropic activity, it is possible that some species present in resin have already disappeared.

7) line 25 should read: However, age and geographical origin … - replaced

8) line 27: replace importance by scientific value - replaced

9) lines 36 and 42: replace hottest hotspot by a meaningful term

We consider it do not to proceed. The term “hottest hotspots” was defined by Myers et al. (2000) in Nature for the eight unusual rich hotspots on biodiversity in the world, and Madagascar is one of the so-called “hottest hotspot”.

Myers et at. (2000). Biodiversity hotspots for conservation priorities. Nature 403: 853–858.

10) line 45: geographically isolated – we are included the term “geographically”

11) line 90: replace a problem by problematic – we are replaced “problem” by “ambiguous” as the Editor suggested.

12) line 333: replace coal by charcoal - replaced

13) line 348: University of Barcelona. Where exactly are the samples housed (department, collection, museum?).

We are included in the text a more precise location of the samples.

14) line 379: most likely it came from the area of Mananjary. How do you know? Speculation only?

Yes, it is not sure the precedence of these pieces. However, in the Mananjary region there is a large current resin trade. Resin is collected in this area for sale as "Madagascar copal" and it is relatively close to the city of Antsirabe (almost 150 km away), at least, much closer than the Sambava region that are almost 1000 km away. It is for this reason that we assume that the material studied comes from Mananjary, and not from the north of Madagascar, the other area where resin is collected for sale.

15) line 395: replace originated by produced - replaced

16) line 479: The coastal forest harbors a very unusual arthropod community - In what manner is it unique?

Madagascar's east lowland forest degrades at high speed because it is one of the most populated areas, but at the same time it is one of the least studied since research teams focus their studies on the rainforest, where there is a greater diversity of flora and fauna. The fauna of arthropods that live in this area is quite different from what is usual to visualize in Malagasy entomological treatises.

17) line 663: I miss reference to some more recent literature on resinicolous fungi such as Beimforde et al. (2017, Arthropod-Plant Interactions), Rikkinen & Schmidt (2018, in Krings et al. Transformative Palaeontology), and resinicolous bacteria (Saint Martin & Saint Martin 2017, Comptes Rendus Palevol).

Any of these references do not refer or have relation to the copal of Madagascar. For this reason, we prefer not included them in the manuscript.

18) line 748: little age variation. 80 to 300 years may appear as large age variation to some readers.

We modified the sentence in order to precise the age: It has a relatively small temporal variation in its age (-80 to -300 years), in relation to the previous proposals that postulate between tens and millions of years.

19) lines 775-778: Did the authors have the opportunity to observe this collection of modern resin from the trees or is this statement based on local hearsay?

Resin harvesting is common in the regions of Mananjary (Nosy Varika, 2013) or Sambava (Andranotsara, 2017) where the people who collect resin from *Hymenaea verrucosa* came to offer us - pieces containing insects. It is true that we had a Malagasy guide who encouraged to the locals. Local people offered resin amounts containing stalactite-shaped pieces over 50 cm long plenty of insect inclusions; however, our affirmation is not based on local hearsay, but direct observation in the field. We also noticed in the field that the material present in the forest soils is poorly preserved. The pieces sold in the markets have not been polished, as we were informed, but they were collected directly from the trees. *Hymenaea* resin polymerizes within a few days and remains transparent until its burial. We reiterate that pieces sold as polished "copal" are fresh resin directly collected from the trees without preparation.

19) line 866: Madagascar is not a habitat! The island contains many habitats, most of them are critically endangered.

The word "in" has been erased. The sentence refers to the lowland coastal forest area on the Eastern Madagascar, as one of the most degraded habitats of the island.

20) The manuscript would greatly benefit from a thorough improvement of the language.

The manuscript was reviewed by an English researcher before first submission to the journal. However, before resubmission the new version to PlosOne was corrected by the Language Services of the University of Barcelona, in order to improve the English.

**Reviewer #2:**

The paper would benefit from a few modifications. Some comments and suggestions are made in the attached manuscript annotated. A few general comments:

1) In my opinion, the title is not reflecting accurately the main focuses of the article defined in the introduction (age and geographical origin of the “Madagascar copal”). Based on the title, I was initially expecting studies of the fauna trapped in the resin, and estimations of the loss of biodiversity in the Anthropocene.

We replaced the title exactly as suggested the reviewer #1.

2) Unnecessary information. For example: the section Copal as a trade product. I am not convinced about the utility of so much archaeological details for answering your research questions. I would delete most of it, if not all (maybe keep some very basic information and move it to the introduction).

We consider the historical information we provide in order to differentiate the moment when the trade of the "Madagascar copal" is started to be individualized with respect to the "Zanzibar copal" or "East African copal" it is essential to know from what date we can carefully differentiate the origin of the historical material housed in museums. However, this part has been summarized leaving what we considered more essential.

A few problems of writing style requiring some editing. Some suggestions/corrections are given in the attached manuscript.

All these changes have been accepted. Also, some sentences have been rewriting, in order to improve understanding.

3) The term “Recent” is frequently used throughout the manuscript to designate non-fossil taxa. I would favor the term “modern” instead. –

The use of the terms “Recent” or “modern” in the text have been reviewed by the correction service of the University of Barcelona, in order to be used each word in its place properly.

4) The use of quotation marks breaks up the text unnecessarily. It may be better to only use quotation marks around terms like “Madagascar copal” once, at the first mention of the term, and delete all subsequent uses.

If it is not required by the Journal or the Editor, we consider that quotation marks must be kept because the name “Madagascar copal” is mention as a literal name through the whole manuscript. Please, note that the main conclusion of this manuscript is that it is not copal, thus we prefer to maintain that notation depending of the progress of the manuscript content and the contexts in each case. In respect to this, we included these quotation marks very carefully in the original manuscript.
